# Estimating the Impact of Tuberculosis Case Detection in Constrained Health Systems: An Example of Case-Finding in South Africa

**DOI:** 10.1093/aje/kwz038

**Published:** 2019-03-02

**Authors:** Thomas Sumner, Fiammetta Bozzani, Don Mudzengi, Piotr Hippner, Rein M Houben, Vicky Cardenas, Anna Vassall, Richard G White

**Affiliations:** 1 TB Modelling Group, TB Centre, Centre for the Mathematical Modelling of Infectious Disease, Department of Infectious Disease Epidemiology, London School of Hygiene and Tropical Medicine, London, United Kingdom; 2 Department of Global Health and Development, London School of Hygiene and Tropical Medicine, London, United Kingdom; 3 Aurum Institute, Johannesburg, South Africa

**Keywords:** health resources, mathematical models, South Africa, tuberculosis

## Abstract

Mathematical models are increasingly being used to compare strategies for tuberculosis (TB) control and inform policy decisions. Models often do not consider financial and other constraints on implementation and may overestimate the impact that can be achieved. We developed a pragmatic approach for incorporating resource constraints into mathematical models of TB. Using a TB transmission model calibrated for South Africa, we estimated the epidemiologic impact and resource requirements (financial, human resource (HR), and diagnostic) of 9 case-finding interventions. We compared the model-estimated resources with scenarios of future resource availability and estimated the impact of interventions under these constraints. Without constraints, symptom screening in public health clinics and among persons receiving care for human immunodeficiency virus infection was predicted to lead to larger reductions in TB incidence (9.5% (2.5th–97.5th percentile range (PR), 8.6–12.2) and 14.5% (2.5th–97.5th PR, 12.2–16.3), respectively) than improved adherence to diagnostic guidelines (2.7%; 2.5th–97.5th PR, 1.6–4.1). However, symptom screening required large increases in resources, exceeding future HR capacity. Even under our most optimistic HR scenario, the reduction in TB incidence from clinic symptom screening was 0.2%–0.9%—less than that of improved adherence to diagnostic guidelines. Ignoring resource constraints may result in incorrect conclusions about an intervention’s impact and may lead to suboptimal policy decisions. Models used for decision-making should consider resource constraints.

While South Africa has experienced a decline in tuberculosis (TB) notifications from a peak in 2011 (1), TB remains a major public health problem. In 2017, there were an estimated 322,000 incident cases of TB in South Africa (2) and TB was the leading infectious cause of death (3). Of the estimated incident cases in 2017, authorities were notified of fewer than 70%, highlighting the need for improved TB case-finding.

Diagnosis of TB in South Africa has traditionally relied on passive presentation to health-care services, leading to delays in diagnosis, especially among persons not infected with human immunodeficiency virus (HIV) (4); this suggests that new approaches are required to identify people with TB sooner. Several studies have identified missed opportunities for TB screening in persons already visiting public health clinics (5, 6), and recent initiatives in South Africa have led to increases in screening of public health clinic patients. Mathematical modeling (7) has suggested that increased clinic-based screening, a form of intensified case-finding (ICF), could result in significant reductions in TB incidence. However, economic analysis suggests that ICF, while cost-effective, is likely to require large financial commitments (8, 9).

Traditionally, estimates of the impact and cost-effectiveness of TB case detection (and other interventions) have assumed that the only constraint on scale-up is the health sector budget. However, within-sector budgets (e.g., the TB program budget) may also be constrained if policy-makers are unwilling to disinvest in other areas. It is also important to consider constraints on human resources (HR) and other health system requirements. Even if funding is available, it may take time to produce the necessary staffing and infrastructure to deliver services. Financial and health systems constraints may limit the impact of proposed interventions, either because the desired level of coverage cannot be achieved on the anticipated time scale or because resources must be reallocated from elsewhere to achieve it.

While mathematical models are increasingly being used to predict the impact of TB control strategies and to inform policy-making, few models include resource constraints. Instead, models assume some intervention coverage and estimate the costs and impact of achieving this coverage, assuming sufficient health system capacity. This issue has recently been highlighted in HIV modeling, with Mikkelsen et al. (10) calling for new approaches to dynamically incorporate constraints into mathematical models of the scale-up of antiretroviral therapy (ART). Lin et al. (11) and Langley et al. (12) have addressed the issue of health system capacity in TB modeling using an approach that links detailed epidemiologic and operational models. However, this approach requires a detailed understanding of the flow of patients through the health system, and linking the two models has proven technically challenging (12).

We propose an alternative pragmatic approach for incorporating resource constraints, illustrated via application to ICF for TB in South Africa. The approach was developed and applied in the context of informing the South African National Strategic Plan for HIV, Tuberculosis, and Sexually Transmitted Infections (13) and was conducted within the tight time frame that national planning processes allow (14).

Using a dynamic transmission model, secondary data from costing studies, and national data on health sector HR, we estimated the potential epidemiologic impact and financial and nonfinancial resource requirements of achieving prespecified coverage targets for 9 ICF interventions. We then estimated the coverage and impact that could be achieved if resource use were to remain within estimates of future capacity.

## METHODS

### Transmission model

The TB transmission model used is similar to a number of published TB models (15, 16), with additional refinements to describe screening and diagnosis in South Africa. Full details are given in Web Appendix 1, Web Tables 1–14, and Web Figures 1–4 (available at https://academic.oup.com/aje). The model was used to project the impact of ICF strategies in South Africa from 2016 to 2035.

In the model, the population is divided into 3 TB states: susceptible, latently infected, and active disease (stratified into smear-positive and smear-negative states). Susceptible persons are infected at a rate that depends on the prevalence of active disease. Following infection, some proportion of infected persons progress directly to active disease; the remainder enter the latent state. Latently infected persons may remain infected, progress to disease (reactivation), or be reinfected. Persons with active disease can self-cure, die, or be diagnosed and treated for TB. Each of the infection and disease states is further stratified by treatment history (previously treated or treatment-naive) and drug resistance status (susceptible or multiple-drug-resistant).

The model includes HIV infection status and the association of coinfection with the risk of developing and dying from TB. Age-specific HIV incidence and ART coverage are external inputs to the model. The HIV-infected population is stratified by CD4 cell count and time on ART. ART is assumed to reduce the risk of developing TB and the risks of HIV- and TB-associated mortality.

Screening, diagnosis, and treatment are a simplification of the national TB diagnostic guidelines in South Africa (17) (Web Figures 2 and 3). These are not included as explicit states in the model. Instead we calculate the rate at which people start treatment based on the steps of the diagnostic pathway (see Web Appendix 1 for details).

### Base case and interventions

The base case described continuation of current TB care in South Africa. We assumed that people self-presented (passively) with symptoms suggestive of TB at rates estimated by fitting the model to incidence and notification data from the period prior to the introduction of ICF. We estimated current rates of ICF (among clinic patients and HIV-infected persons enrolled in care) by fitting the model to the total reported number of persons screened for TB in recent years. Among persons not receiving HIV care, we assumed that those presenting with prolonged cough (of more than 2 weeks) were referred for sputum testing (17), while those in HIV care were evaluated on the basis of the presence of any TB symptoms (using the World Health Organization (WHO) screening tool). On the basis of data reported to WHO, we assumed that 40% of persons enrolled in HIV care had been asked about TB symptoms at their last visit (18). National guidelines also recommend that household contacts of TB cases be screened for TB. We assumed that visits by contacts to TB screening clinics were captured in the baseline passive screening rate. Active contact investigations involving household visits are not widely implemented in South Africa and were not included in the model.

Eighty percent of initial diagnostic tests were assumed to be carried out via the Xpert MTB/RIF assay (Cepheid Inc., Sunnyvale, California), which detects *Mycobacterium tuberculosis* (MTB) complex and rifampin (RIF) resistance, with the remainder being conducted via smear microscopy (13). Xpert is a nucleic acid amplification test that was endorsed by WHO in 2013 and subsequently adopted as the initial test for TB diagnosis in South Africa. HIV-infected persons with a negative Xpert test result should have further sputum samples collected for culture and drug susceptibility testing, in line with national guidelines (17). However, on the basis of data from the Xpert for Tuberculosis: Evaluating a New Diagnostic (XTEND) Study, we assumed that only 14% of patients received appropriate follow-up (19). On the basis of a systematic review published in 2014 (20) and data from the XTEND Study (21), we assumed pretreatment loss to follow-up of 17%. Historical values for treatment success were based on national treatment outcome data: 78% for drug-susceptible TB and 50% for multiple-drug-resistant TB in 2015 (22). Full details on the base-case assumptions can be found in Web Appendix 1.

In a secondary analysis, we adapted the base case to include the following activities, planned in South Africa as part of the National Strategic Plan for HIV, Tuberculosis, and Sexually Transmitted Infections (13): reducing pretreatment loss to follow-up by 80% (from 17% to 4%) by 2021 and introducing short-course multiple-drug-resistant TB treatment (23) alongside continued use of bedaquiline for pre–extensively drug-resistant and extensively drug-resistant TB (24). This analysis allowed us to explore how the impact and resource use of the ICF strategies may be altered by other future improvements in the TB program.

We considered 9 interventions representing increased adherence to current diagnostic guidelines and various strategies for ICF. These were defined in collaboration with policy-makers as part of the South African TB Think Tank Project (14) and are shown in Table 1. For all interventions, we assumed linear scale-up to the target values between 2017 and 2021.

**Table 1.** Summary of the Interventions included in a model of tuberuculosis transmission, South Africa, 2016–2035^a^

Intervention	Description
1. Base case	Continuation of current practice
2. Xpert^b^ testing	Use of Xpert as the first-line test is increased from 80% to 100%.
3. Guidelines	Adherence to Xpert-negative guidelines is increased from 14% to 90% in persons known to be infected with HIV.
4. 2 + 3	Combination of interventions 2 and 3
5. Cough HIV+	Cough-based screening (compared with WHO symptom screening in the base case) in 100% (compared with 40% in the base case) of HIV-infected persons enrolled in care
6. Cough PHC	Cough-based screening in 90% (compared with 50% in the base case) of PHC patients
7. Symptom HIV+	WHO symptom screening in 100% (compared with 40% in the base case) of HIV-infected persons enrolled in care
8. Symptom PHC	WHO symptom screening (compared with cough-based screening in the base case) in 90% (compared with 50% in the base case) of PHC clinic patients
9. 4 + 6	Combination of interventions 2, 3, and 6
10. 4 + 8	Combination of interventions 2, 3, and 8

Abbreviations: HIV, human immunodeficiency virus; HIV+, human immunodeficiency virus–positive; MTB, *Mycobacterium tuberculosis*; PHC, public health clinic; RIF, rifampin; WHO, World Health Organization.

^a^ Interventions were scaled up linearly from 2017.

^b^ Xpert MTB/RIF assay (Cepheid Inc., Sunnyvale, California).

### Resource requirements and constraints

To illustrate our approach, we considered 3 types of resources and their future constraints: budget (total costs of the TB program), HR (amount of nurse time spent on TB activities), and diagnostic (ratio of number of Xpert tests to number of TB notifications). These were identified through discussions with local stakeholders that took place as part of the South African TB Think Tank Project (14). During these discussions, stakeholders from the South African National Department of Health highlighted financial, HR, and diagnostic supplies constraints as critical areas to be addressed in this analysis. Full details on the methods used to estimate unit costs and nurse time and the derivation of the future constraints can be found elsewhere (25).

Three future scenarios for the budget and total nurse time were considered (Table 2). We refer to these as the low (most restrictive), medium, and high (least restrictive) scenarios; further details are provided below. For the diagnostic constraint, we considered a single scenario in which the ratio of Xpert tests to TB notifications was capped at 20:1. This constraint reflects a limit on diagnostic supplies (Xpert cartridges) purchased annually in South Africa. The ratio was set following discussion with the South African National Department of Health.

**Table 2.** Resource Constraints Applied in a Model of Tuberculosis Transmission, South Africa, 2016–2035

Type of Constraint	Constraint Scenario^a^		
	Low	Medium	High
Budget (total cost of TB program)	GDP growth	Low scenario plus reprioritization based on disease burden from 2017 to 2021	Medium scenario plus earmarked taxes from 2017 to 2021
HR (amount of nurse time spent on TB activities)	Population growth	Low scenario plus reprioritization based on disease burden from 2017 to 2021	Medium scenario plus historical growth in nursing workforce
Diagnostic^b^ (ratio of no. of Xpert^c^ tests to no. of TB notifications)	Xpert test:notification ratio does not exceed 20:1	Xpert test:notification ratio does not exceed 20:1	Xpert test:notification ratio does not exceed 20:1

Abbreviations: GDP, gross domestic product; HR, human resources; MTB, *Mycobacterium tuberculosis*; RIF, rifampin; TB, tuberculosis.

^a^ The low scenario is the most restrictive; the high scenario is the least restrictive.

^b^ A single diagnostic constraint scenario was considered.

^c^ Xpert MTB/RIF assay (Cepheid Inc., Sunnyvale, California).

The low-budget (most restrictive) scenario was based on predicted gross domestic product growth of 1.7% per year (26). The medium scenario additionally assumed that a proportion of the current budget was reallocated to TB in line with the proportion of deaths in South Africa attributable to TB (approximately 10%–15%), and the high scenario assumed that a proportion of a health budget that achieved its full fiscal space growth was similarly reallocated.

In the low-HR scenario, the number of nurse minutes spent on TB was adjusted in future years on the basis of population growth only. The medium constraint incorporated a reallocation of the current workforce to TB on the basis of disease burden, as above; and the high scenario similarly reallocated a proportion of the nursing workforce that achieved its maximum growth on the basis of historical growth rates.

The budget and nurse time required in the base case and in each intervention were calculated by multiplying unit costs and minutes of nurse time per activity by the outputs of the transmission model (Table 3). Use of Xpert testing was calculated by dividing the number of tests (an output of the model) by the model-estimated number of notifications.

**Table 3.** Amounts of Required Nurse Time and Costs of Various Tuberculosis Control Activities, per Unit of Activity, South Africa, 2016

Activity	Nurse Time, minutes	Cost, $US	Unit of Activity
Passive screening	2.63	0.68	Per screen
Cough screening	1.26	0.68	Per screen
WHO symptom screening	4.00	1.36	Per screen
Sputum smear microscopy	3.16	10.87	Per screen
Xpert^a^ testing	3.16	32.24	Per screen
Follow-up of Xpert-negative persons	8.61	24.00	Per screen
First-line treatment (initiation phase, 2 months)	35.72	21.43	Per month^b^
First-line treatment (continuation phase, 4 months)	7.57	21.43	Per month^b^
MDR treatment, DOT (initiation phase, 6 months)	237.04	359.10	Per month^b,c^
MDR treatment, non-DOT (initiation phase, 6 months)	84.47	359.10	Per month^b,c^
MDR treatment, DOT (continuation phase, 18 months)	159.83	359.10	Per month^b^
MDR treatment, non-DOT (continuation phase, 18 months)	84.47	359.10	Per month^b^
Isoniazid preventive therapy	5.54	7.81	Per month

Abbreviations: DOT, directly observed therapy; MDR, multiple-drug-resistant; MTB, *Mycobacterium tuberculosis*; RIF, rifampin; WHO, World Health Organization.

^a^ Xpert MTB/RIF assay (Cepheid Inc., Sunnyvale, California).

^b^ On the basis of discussion with the South African National Department of Health, we assumed that 20% of drug-susceptible patients and 20% of decentralized MDR patients receive treatment via DOT; the remainder only visit clinics monthly to obtain antituberculosis medication.

^c^ Sixty percent of MDR patients are hospitalized during the intensive phase. This activity is not included in our estimates of public health clinic nurse time.

The total costs, nurse time, and Xpert test:TB notification ratio required by each intervention were compared with the constraint scenarios to identify interventions that exceeded the constraints. These interventions were then resimulated to estimate the epidemiologic impact under the constrained scenarios. For the budget and HR constraints, we iteratively reduced the maximum intervention coverage achieved in 2021 (assuming linear scale-up from 2017, as in the unconstrained scenario) such that the projected cost or nurse time remained below the constraint over the entire time horizon (2017–2035). When implementing the constraint on the Xpert test:TB notification ratio, we assumed that an intervention would be stopped (coverage reduced to zero) when the 20:1 ratio suggested by the National Department of Health was exceeded.

### Model calibration

The model was fitted to TB notification data (total and multiple-drug-resistant) (18), the number of screenings reported to the National Department of Health, the number of laboratory tests conducted (27), and estimated TB incidence and mortality (1). Details can be found in Web Appendix 2 and Web Table 15. In summary, values for parameters were sampled from prior specified ranges. The model was then calibrated in a 2-step process by varying selected model parameters to minimize the weighted sum of squared differences between the model and the observed data. First, the contact rate and passive screening rate were varied to match the incidence and notifications in 1990 (before the increase in HIV-associated TB). Secondly, the increase in passive screening, the rate of ICF, the rate of acquisition of drug resistance, a multiplier for the impact of ART on TB risk among people living with HIV, and a multiplier for the TB mortality rate in HIV-infected persons were varied to fit the model to all calibration data from 1990 to 2015. This process was repeated 1,000 times to incorporate the uncertainty in the unfitted parameters.

## RESULTS

### Baseline fit and base-case projection

**Figure 1.**
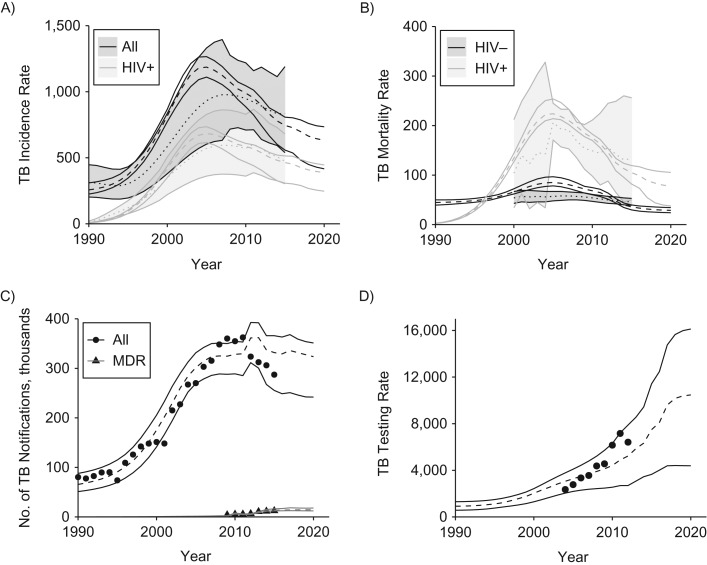
Baseline fit of a tuberculosis (TB) transmission model to TB data from South Africa, 2016–2035. A) TB incidence per 100,000 population, overall (dark gray) and among persons positive for human immunodeficiency virus (HIV+) (light gray). The dotted line shows the point value, and the shaded area shows the range of the World Health Organization estimate. The dashed line shows the median value, and the solid lines show the range of the model output. B) TB mortality per 100,000 population in HIV-uninfected persons (HIV−; dark gray) and people living with HIV (HIV+; light gray). Other details are the same as those for panel A. C) Numbers of TB notifications (in thousands) for all forms of TB (circles) and multiple-drug-resistant (MDR) TB (triangles). Points show the reported data. The dashed line shows the median value, and the solid lines show the range of the model output. D) Rate of laboratory testing for TB per 100,000 population. Other details as the same as those for panel C.

**Figure 2.**
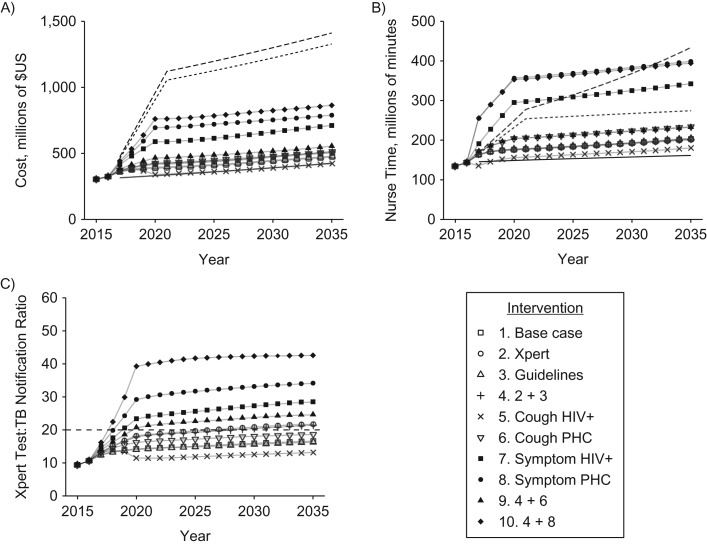
Model projection of future costs, human resource requirements, and Xpert test:tuberculosis (TB) notification ratio (ratio of number of Xpert tests (Xpert MTB/RIF assay; Cepheid Inc., Sunnyvale, California) to number of TB notifications) of the TB control program in South Africa, 2016–2035. Symbols show the median model prediction for each intervention from 2016 to 2035. A) Total costs of TB control activities, in millions of US dollars; B) nurse time spent on TB activities, in millions of minutes; C) Xpert:notification ratio. In panels A and B, solid lines show results for the low (most restrictive) constraints, dotted lines show results for the medium constraints, and dashed lines show results for the high (least restrictive) constraints. In panel C, results are shown (dashed line) for only a single constraint (a ratio of 20:1). HIV+, positive for human immunodeficiency virus; MTB, *Mycobacterium tuberculosis*; PHC, public health clinic; RIF, rifampin.

The model reproduces the trends in TB incidence, mortality, and notifications and predicts continued declines in incidence and mortality up to 2020 (Figure 1). Future notifications remain largely constant, although with large uncertainty. Short-term increases in notifications result from an increase in the number of true-positive TB cases diagnosed. However, the model indicates that over time, as TB incidence falls, there will be an increasing number of false-positive notifications due to the imperfect specificity of the diagnostic process. Reported testing volumes are within the model uncertainty. Baseline estimates of the total cost and nurse minutes spent on TB activities (see Figure 2) in 2015/2016 are consistent with previous estimates (25).

Total costs, nurse minutes, and the ratio of Xpert tests to TB notifications are predicted to increase over time. The increases in costs and nurse time are driven by population growth and increased access to HIV care, both of which result in increased numbers of people being screened for TB. The increase in the Xpert test:notification ratio is driven by the fall in the prevalence of TB; as TB becomes rarer, the number of persons who will have to be tested in order to find 1 TB case will increase.

### Impact of interventions—unconstrained scenario

**Figure 3.**
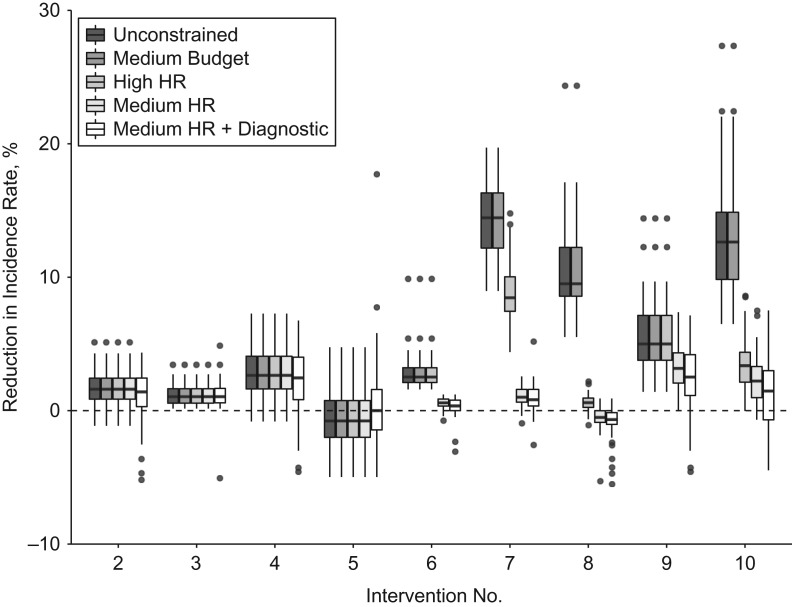
Reductions in tuberculosis (TB) incidence predicted by a TB transmission model, South Africa, 2016–2035. The graph shows the percentage reduction in the TB incidence rate in 2035 as compared with baseline in 2035 (intervention 1). Shading indicates the type of constraint applied to the model. Boxes show the 25th–75th percentile range, whiskers indicate 1.5 times the interquartile range, and black circles show outliers. The high (least restrictive) budget constraint is not shown because results were the same as those for the medium budget constraint. Values above 0 (dashed horizontal line) indicate a larger reduction in TB incidence as compared with baseline. HR, human resources.

In the base case (which includes current levels of ICF), the predicted reduction in TB incidence from 2016 to 2035 is 18.9% (2.5th–97.5th percentile range (PR), 3.5–29.4). Figure 3 and Table 4 show the additional percentage change in the incidence rate in 2035 compared with the base case (intervention 1) for each intervention. When not accounting for constraints (darkest bars), the largest reductions in incidence are predicted for interventions which include increased screening coverage using the WHO symptom tool (interventions 8 (9.5%; 2.5th–97.5th PR, 8.6–12.2), 10 (12.6%; 2.5th–97.5th PR, 9.8–14.9), and 7 (14.5%; 2.5th–97.5th PR, 12.2–16.3), in order of increasing impact). A smaller but significant reduction (5.0%; 2.5th–97.5th PR, 3.8–7.1) is also predicted for increased use of cough-based screening in public health clinic patients when combined with improved adherence to the diagnostic algorithm (intervention 9).

**Table 4.** Reductions in Tuberculosis Incidence Predicted by a Tuberculosis Transmission Model, South Africa, 2016–2035

Intervention^a^	Median (2.5th–97.5th PR) Reduction in TB Incidence, %^b^
1. Base case	0 (0–0)
2. Xpert^c^ testing	1.6 (0.9–2.4)
3. Guidelines	1.1 (0.6–1.6)
4. 2 + 3	2.7 (1.6–4.1)
5. Cough HIV+	−0.7 (−2.0–0.76)
6. Cough PHC	2.6 (2.1–3.2)
7. Symptom HIV+	14.5 (12.2–16.3)
8. Symptom PHC	9.5 (8.6–12.2)
9. 4 + 6	5.0 (3.8–7.1)
10. 4 + 8	12.6 (9.8–14.9)

Abbreviations: HIV+, human immunodeficiency virus–positive; MTB, *Mycobacterium tuberculosis*; PHC, public health clinic; PR, percentile range; RIF, rifampin; TB, tuberculosis.

^a^ Numbers refer to the interventions listed in Table 1.

^b^ Values indicate the predicted median percent reduction in TB incidence in 2035 in the intervention group compared with the base case (intervention 1).

^c^ Xpert MTB/RIF assay (Cepheid Inc., Sunnyvale, California).

Increased utilization of Xpert testing (intervention 2) and improved adherence to diagnostic guidelines among persons with negative Xpert assay results (intervention 3) produce small reductions in incidence. However, the combination of these two strategies (intervention 4) produces an impact similar to that of increases in public health clinic screening (intervention 6). The change to cough-based screening among persons receiving HIV care (intervention 5), which is not recommended in the national or WHO guidelines, results in an increase in incidence compared with the base case. This is because cough-based screening has lower sensitivity than the WHO screening algorithm, which is included in the base case. As a result, despite the assumed increase in coverage, this intervention strategy is inferior to continuation of current practice.

### Resource requirements

Figure 2 shows the predicted resources required for the base case and each intervention from 2016 to 2035.

All interventions, including the base case, exceed the low budget and low nurse time constraints (the most restrictive scenarios); these scenarios were not considered further. In contrast, the medium and high budget constraints are not exceeded by any intervention. For interventions 6–10, which include increases in ICF, the model predicts increases in nurse time which exceed the proposed constraints. This is particularly the case for interventions based on WHO symptom screening (interventions 7, 8, and 10) because of the increased amount of time taken to carry out the screening and the lower specificity as compared with cough screening, which results in an increased number of tests being administered in persons without TB. The Xpert constraint is exceeded by interventions that include increased use of symptom screening (interventions 7, 8, and 10) and strategies that include increased use of Xpert as the first-line test (interventions 2, 4, and 9). The most ambitious intervention (intervention 10) requires more than 45 Xpert tests per TB notification.

### Impact of interventions—constrained scenarios

Inclusion of medium budget constraints in the model (Figure 3, dark gray bars) does not change the predicted impact, because the future budget predictions do not exceed these constraints. The high budget constraint is also not exceeded (not shown). Similarly, the impact in the base case (intervention 1), strategies focused on improved adherence to guidelines (interventions 2–4), and the use of cough screening in HIV-infected persons (intervention 5) are not affected by the nurse time constraint.

Both high (Figure 3, medium gray bars) and medium (Figure 3, light gray bars) HR constraints reduce the impact of increased ICF (interventions 6–10), because the previously assumed coverage (see Table 1) cannot be reached. Under the high HR constraint, the largest median impact is still observed for increased symptom screening of HIV-infected persons (intervention 7). However, for the medium HR constraint, the ranking of the interventions is changed, with the largest impacts predicted for increased adherence to guidelines (intervention 4) and the combination with increased cough screening in clinic patients (intervention 9). The additional effect of the Xpert constraint on top of the medium HR constraint (Figure 3, white bars) is small, partly because much of the impact in each intervention has been achieved by the point at which the Xpert test:notification ratio exceeds 20:1.

### Secondary analysis

The results for the secondary analysis including strategy outlined in the National Strategic Plan for HIV, Tuberculosis, and Sexually Transmitted Infections are qualitatively the same. In the base case, the model predicts a greater reduction in incidence due to the additional impact of improved linkage to care. However, the incremental benefit of each intervention, when combined with reduced pretreatment loss to follow-up, is smaller since the impacts are not additive. The overall costs and nurse time are lower in the National Strategic Plan scenario than for the corresponding interventions in the primary analysis; however, the same interventions exceed the constraints. Web Appendix 3 and Web Figures 5–7 provide further details on the results of the secondary analysis.

## DISCUSSION

Our results show that ICF may result in significant reductions in TB incidence, but with large increases in financial and HR requirements. While the costs may remain below the assumed budget projections, ICF strategies may exceed HR capacity, even under optimistic scenarios of reallocation of nurse time to TB and normative limits around Xpert test numbers. When the HR constraint is included in the model, the ranking of interventions by impact is changed. In particular, the impact of ICF strategies is greatly reduced and those strategies may, in rare cases, be less effective than continuation of current practice.

Our approach was developed and implemented in the context of supporting the use of modeling to inform TB control policy in South Africa (14). Our aim was to demonstrate the feasibility of a simple approach to highlight the potential importance of constraints. In this light, several simplifying assumptions were made. When incorporating constraints, we assumed that other TB control activities would continue at their current levels and that the coverage of the new intervention would be reduced to satisfy the constraint. An alternative would be to consider that the full intervention coverage is reached and that the capacity for other activities must be reduced to compensate. This approach could be considered in the framework used here but would require rules for prioritization of activities.

Our illustrative approach also assumed a single change to intervention coverage to satisfy the constraints over the entire time period considered. In reality, the mixture and coverage of interventions may change over time as capacity varies. In our example, the HR constraint was typically exceeded in the short term before the HR capacity could be increased. As such, it is possible that coverage could subsequently be increased in the future. Our results are therefore probably a conservative estimate of the impact that could be achieved under these constraints. Yaesoubi and Cohen (28) modeled dynamic case-finding policies and showed that they were preferable to static policies. However, they only considered the financial requirements of those policies. Combining these two approaches may be a useful way to identify strategies that account for the time scales of staff recruitment.

Several studies have addressed the issue of resource constraints in infectious disease models. Lin et al. (11) proposed an alternative approach with which to incorporate health system capacity in models by combining a discrete event simulation of the health system with a compartmental model of TB transmission. This approach has been used successfully to explore the impact of new diagnostics in Tanzania (12); however, it requires a detailed understanding of the whole health system in a country and presents computational demands in linking the models. Hontelez et al. (29) incorporated constraints into their analysis of changes to HIV treatment eligibility by defining a priori a set of treatment scale-up scenarios that represented potential supply- and demand-side constraints. In contrast, our approach is similar to the AsiaFluCap Simulator used in the planning of pandemic influenza control (30); the outputs of the model are used to dynamically estimate the resource needs of different interventions and compare these with constraints defined using a combination of secondary data and policy-maker consultation.

While we attempted to include the key aspects of TB control in South Africa, there were several important limitations. We relied on routinely collected data, which may be subject to inaccuracy and bias. We assumed that ICF occurs independently of true TB status and hence may underestimate the TB prevalence in the population screened and the ICF impact. We also assumed that linkage to TB treatment and completion of treatment are equal in persons identified passively and persons identified via ICF. If those identified via ICF are less likely to start treatment (31), the model may overestimate the impact and resource use of the ICF interventions. It is also possible that linkage to TB treatment may be greater for people receiving HIV care than for people not engaged with the health system. If this were the case, it might increase the relative impact of screening among people living with HIV as compared with other forms of ICF.

In this work, we considered South Africa as a whole. The resource requirements of ICF could be reduced by targeting interventions toward geographical regions with the highest burden. The preferred strategies may differ between areas because of differences in factors such as the prevalence of HIV coinfection.

The model includes the association between HIV infection and risk of TB but does not currently include other known risk factors for TB, such as diabetes or smoking. Strategies for detecting and preventing TB in these risk groups may form an important part of future efforts to control TB in South Africa. Strategies that target these and other risk groups may reduce the resource requirements of case-finding interventions.

Our results can be used to illustrate to policy-makers the need to define policies that expand TB services at the same time as they address constraints to expansion. For example, in this work we assumed that all activities were conducted by nurses, the main cadre of staff that have historically been responsible for delivering TB services in South Africa. Our findings show that policies which aim to increase TB screening must be accompanied by strategies to address HR constraints, via investment in training or by task-shifting activities like contact tracing or symptom screening to other staff, such as lay workers. While none of the interventions exceeded the medium and high budget constraints, the constraint scenarios assume a substantial reallocation of the health budget. Without this reallocation, none of the interventions are feasible. Limiting use of Xpert testing based on the number needed to test to diagnose 1 TB case may also restrict the reduction in incidence that can be achieved. As TB burden declines, this ratio will need to increase to ensure that cases are not missed.

It is also important to consider how these resource constraints may affect the cost-effectiveness of different strategies. Strategies that are deemed cost-effective in the absence of constraints may not be cost-effective if the potential impact is limited by constraints or the costs of relaxing constraints are included in the analysis. This is the focus of ongoing research.

In conclusion, this work highlights the importance of including resource constraints in models used to evaluate TB control strategies. Ignoring these constraints may result in overestimation of the achievable impact of interventions in the absence of other actions to increase health system capacity, and ultimately may lead to inappropriate or unwarranted policy decisions.

## Supplementary Material

Web Material

## Abbreviations

ART: antiretroviral therapy
HIV: human immunodeficiency virus
HR: human resources
ICF: intensified case-finding
PR: percentile range
TB: tuberculosis
XTEND: Xpert for Tuberculosis: Evaluating a New Diagnostic
WHO: World Health Organization
