# Supplementary material for: Estimating the Impact of Tuberculosis Case Detection in Constrained Health Systems: An Example of Case-Finding in South Africa
Source: Am J Epidemiol. 2019 Mar 2;188(6):1155–64. doi: 10.1093/aje/kwz038 (PMC6545281; doi:10.1093/aje/kwz038)
Supplement: Web Material [file kwz038_sumner_web_material_final.pdf]

WEB MATERIAL

Estimating the Impact of Tuberculosis Case Detection in Constrained Health Systems: An Example of Case-Finding in South Africa

Tom Sumner, Fiammetta Bozzani, Don Mudzengi, Piotr Hippner, Rein M. Houben, Vicky Cardenas, Anna Vassall, and Richard G. White

Contents

Web Appendix 1: Model Details

Section

1.1 TB Model (Web Figure 1, Web Tables 1 and 2)
1.2 HIV Model (Web Tables 3–5)
1.3 Demographic model (Web Figure 2)
1.4 Base-case care and control assumptions (Web Tables 6–11, Web Figures 3 and 4)
1.5 NTP Scenario
1.6 Knowledge of HIV status (Web Tables 12 and 13)
1.7 Model equations (Web Table 14)

Web Appendix 2: Model Calibration

Web Table 15

Web Appendix 3: Additional Results—Secondary Analysis

Web Figures 5–7

Web References

WEB APPENDIX 1

Model Details

The model used in this exercise is similar in structure to a number of published TB models [1, 2] with additional refinements to describe diagnostic processes. The model is age structured (by 5-year age groups) and includes HIV and ART.

1.1 TB model

Web Figure 1 (see next page) shows a schematic of the TB model structure. The HIV structure and demographic processes are not included in the figure for clarity. The population is divided into 3 main “TB” states: susceptible (S), latently infected (L), and active disease (stratified into smear-positive (I) and smear-negative (N) states). Susceptible individuals are infected at a rate that depends on contact between individuals and the prevalence of active disease in the population. Following infection, some proportion progress directly to active disease, with the remainder entering the latent state. Latently infected individuals may remain infected, progress to disease (reactivation), be re-infected or incorrectly treated for TB. Individuals with active disease can self-cure, die or be diagnosed and treated for TB. Each of the infection and disease states is further stratified by treatment history (previously treated (p) or treatment naïve (n)), drug resistance status (susceptible (s) or multidrug-resistant (m) (MDR)) and HIV and ART status (see section 1.2). Web Table 1 lists the TB states. Web Table 2 details the natural history parameters used in the model.

(Web Figure 1 and Web Tables 1 and 2 follow.)

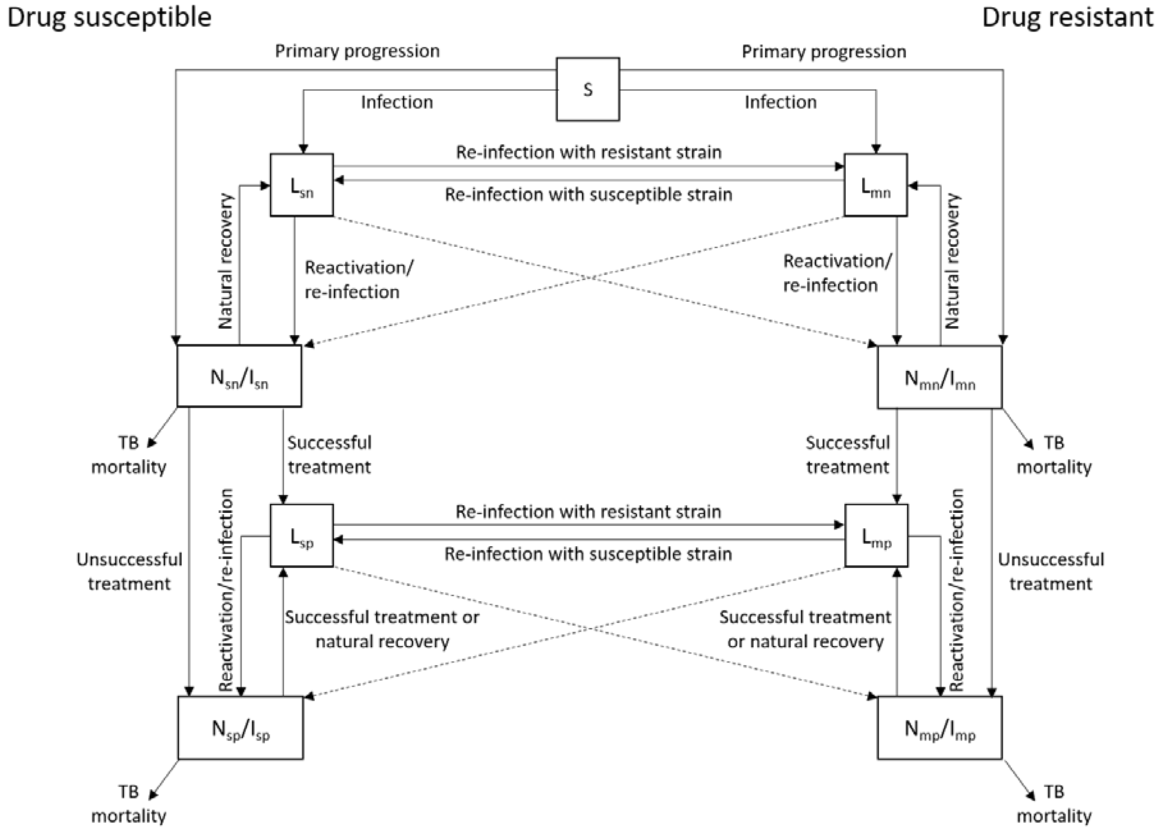

Web Figure 1. Schematic of the TB model structure. For clarity the active disease states (smear-positive (I) and smear-negative (N)) are represented by a single box. Dashed lines indicate direct progression to disease following reinfection with a different strain.

Web Table 1. TB states in the model

Symbol	Description
S	Susceptible
Lsn	Latently infected with drug-susceptible strain, no previous treatment history
Lsp	Latently infected with drug-susceptible strain, previous treatment history
Lmn	Latently infected with drug-resistant strain, no previous treatment history
Lmp	Latently infected with drug-resistant strain, previous treatment history
Nsn	Smear-negative, drug-susceptible TB, no previous treatment history
Nsp	Smear-negative, drug-susceptible TB, previous treatment history
Nmn	Smear-negative, drug-resistant TB, no previous treatment history
Nmp	Smear-negative, drug-resistant TB, previous treatment history
Isn	Smear-positive, drug-susceptible TB, no previous treatment history
Isp	Smear-positive, drug-susceptible TB, previous treatment history
Imn	Smear-positive, drug-resistant TB, no previous treatment history
Imp	Smear-positive, drug-resistant TB, previous treatment history
FTn	Latently infected, incorrectly treated for TB (no previous treatment)
FTp	Latently infected, incorrectly treated for TB (previously treated)

Web Table 2. TB model parameters. In the absence of treatment, the CFR is given by $\mu/(\mu + r)$ and the duration of disease, d by $1/(\mu + r)$ so that μ = CFR/d and $r = (1/d) - \mu$

Parameter	Description	Values	Units	Source
β	Effective contact rate	Fitted	/yr	-
a	Proportion developing primary TB	0.08-0.15	Proportion	[3-5]
v	Reactivation rate	0.001-0.0025	/yr	[3, 5, 6]
p	Protection against TB due to prior infection	0.1-0.63	Proportion	[3]
σ	Proportion developing smear-positive TB, among HIV-uninfected	0.4-0.5	Proportion	[7]
σH	Proportion developing smear-positive TB, among HIV-infected	0.2-0.4	Proportion	[8, 9]
ω	Relative infectiousness of smear-negative TB	0.16-0.32	Proportion	[10, 11]
ϑ	Smear conversion rate among HIV-uninfected	0.007-0.03	/yr	[6, 12]
ϑH	Smear conversion rate among HIV-infected	0.007-0.03	/yr	Assumed same as HIV-uninfected
c_I	Case fatality in untreated smear-positive TB, among HIV-uninfected	0.55-0.75	Proportion	[13]
c_N	Case fatality in untreated smear-negative TB, among HIV-uninfected	0.1-0.3	Proportion	[13]
d	Average duration of untreated TB, among HIV-uninfected	1.5-2.5	yrs	[13, 14]
cH_I	Case fatality in untreated smear-positive TB, among HIV-infected	0.7-0.99	Proportion	[13]
cH_N	Case fatality in untreated smear-negative TB, among HIV-infected	0.63-0.9	Proportion	[13]
dH	Average duration of untreated TB, among HIV-infected	0.25-1	yrs	[13, 14]
μ_I	Mortality rate in smear-positive TB, among HIV-uninfected	c_I/d	/yr	-
μ_N	Mortality rate in smear-negative TB, among HIV-uninfected	c_N/d	/yr	-
r_I	Self-cure rate in HIV-negative, smear-positive	$(1/d)-\mu_I$	/yr	-
r_N	Self-cure rate in HIV-negative, smear-negative	$(1/d)-\mu_N$	/yr	-
μH_I	Mortality rate in smear-positive, HIV-positive	cH_I/dH	/yr	-
μH_N	Mortality rate in smear-negative, HIV-positive	cH_N/dH	/yr	-
rH_I	Self-cure rate in HIV-positive, smear-positive	$(1/dH)-\mu H_I$	/yr	-
rH_N	Self-cure rate in HIV-positive, smear-negative	$(1/dH)-\mu H_N$	/yr	-
f	Relative transmissibility of MDR strains	0.58-0.85	Proportion	[15]
e	Rate of acquisition of MDR	0.01-0.017	/treatment	[16]
g	superinfections	$f/(1+f)$	Proportion	-
H	HIV Incidence	External input	/yr	UNAIDS
m	Migration	External input	/yr	DemProj
μ	Background mortality	External input	/yr	UN population projections 2015 update

1.2 HIV model

The HIV component of the model is similar in structure to AIM (the HIV epidemiology module of Spectrum [17]). The HIV-infected population is divided by CD4 cell count (>500, 350-500, 250-349, 200-249, 100-199, 50-99, <50), and time since ART initiation (no ART, <6 months, 6-12 months, >12 months).

Age stratified HIV incidence, h_i is an external input to the model and is based on UNAIDS estimates for South Africa. New infections are assigned to CD4 categories, *j* based on the age specific distribution of new infections, $\gamma_{i,j}$. It is assumed that HIV incidence is unaffected by TB status. Individuals not on ART progress through CD4 categories at age (i) and CD4 (j) specific rates, $\varepsilon_{i,j}$, calculated from estimated durations of CD4 stage. Those with HIV, but not on ART, are subject to HIV-associated mortality, $\mu H_{i,j}$ which is also assumed to be age and CD4 specific. Values for these parameters are given in Web Table 3.

Historical ART coverage is based on estimates of the number in need of ART (based on eligibility criteria) and number on ART. These values are used to calculate the percentage of the eligible population who should be on ART by age and calendar time. This percentage coverage is used in the model, together with the time dependent CD4 threshold for ART initiation, to calculate the number of people who should be on ART by age. Future ART coverage is assumed to increase in line with UNAIDS projections.

The number of people of age *a* who should be started on ART is calculated as:

$$N_{start} = N_{should} - N_{on} + N_{deaths}$$

That is, the number who start ART (N_{start}) is the difference between the number who should be on ART (N_{should}) and the number who are currently on ART (N_{on}) plus the number who will die while on ART in the current time step (N_{deaths}).

Those starting ART are distributed among the eligible CD4 categories based on the approach used in AIM. This depends on the proportion of the eligible (but not yet on ART) population that are in each CD4 category and the proportion of deaths among those eligible but not on ART occurring in each CD4 category.

$$P_{i,j} = \left(\frac{N_{i,j}}{\sum_{j \in e} N_{i,j}} + \frac{D_{i,j}}{\sum_{j \in e} D_{i,j}}\right)/2,$$

where $P_{i,j}$ is the proportion of new ART initiations of age i who are from CD4 category j, $N_{i,j}$ is the number of individuals of age i who are in CD4 category *j* and not yet on ART, and $D_{i,j}$ is the number of deaths occurring among those age i who are in CD4 category j and not yet on ART. Summations are over the set of CD4 categories, e that are below the eligibility threshold for starting ART.

Mortality rates for HIV-infected individuals on ART ($\mu A_{i,j,l}$) vary by age, CD4 at initiation, time on ART and sex and are shown in Web Tables 4 and 5. As the model does not account for sex, the on-ART mortality rates are weighted by the number of males and females on ART used as input to the model.

In addition to the HIV specific TB model parameters described in section 1.1 the risks of developing TB are affected by HIV in a CD4 dependent manner. Parameters modified in this way are: proportion

developing primary disease following first infection; rate of reactivation; the protection against disease following re-infection due to previous infection.

For each parameter x_i, the CD4 dependent values are given by:

$$x_{i,j}^{H} = x_i RR_1 RR_2^{(500-mid_j)/100}$$

Where superscript H refers to HIV+ not on ART, RR_1 and RR_2 are parameter dependent relative risks and mid_j is the midpoint of CD4 category j (note that the midpoint of the >500 CD4 category is defined as 500 such that $x_{>500} = xRR_1$). The proportion developing primary disease is capped at 1.

ART reverses the impact of HIV on the proportion developing primary disease, the rate of reactivation and the protection provided by prior infection. It also reduces the TB mortality rate compared to the rate in HIV positive individuals not on ART.

For the proportion developing disease, reactivation and mortality the adjusted parameters are given by:

$$x^{A}{}_{i,j,l} = max\left(x^{H}{}_{i,j}(1 - ART_l), x_i\right)$$

where superscript A refers to HIV+ on ART, j is CD4 at time of ART initiation (not CD4 progression is not modelled in those on ART) and l is time on ART. ART_l is the protective effect of ART by time on ART (ART_l can differ for disease and mortality) and the max ensures that ART does not reduce the risks below those experienced by HIV-uninfected individuals of age (x_i).

ART increases the protection provided by prior infection (which is not age dependent) and is implemented as follows:

$$x^{A}{}_{j,l} = min\left(1 - (1 - x^{H}{}_{j}(1 - ART_l), x\right)$$

(Web Tables 3–5 follow.)

Web Table 3. HIV model parameters

				Age Group, years			
**CD4**	**0-4**	**5-9**	**10-14**	**15-24**	**25-34**	**35-44**	**45+**
Distribution of new HIV infections (%), γ							
**>500**	64.3	64.3	64.3	64.3	60.7	58.5	55.2
**350-500**	35.7	35.7	35.7	35.7	39.3	41.5	44.8
**250-349**	0	0	0	0	0	0	0
**200-249**	0	0	0	0	0	0	0
**100-199**	0	0	0	0	0	0	0
**50-99**	0	0	0	0	0	0	0
**<50**	0	0	0	0	0	0	0
Rates of progression through CD4 categories ($years^{-1}$), ε							
**>500**	0.298	0.298	0.298	0.117	0.147	0.183	0.213
**350-500**	0.239	0.239	0.239	0.223	0.240	0.355	0.535
**250-349**	0.183	0.183	0.183	0.294	0.452	0.581	0.855
**200-249**	0.183	0.183	0.183	0.508	1.087	1.250	1.818
**100-199**	0.130	0.130	0.130	0.214	0.637	0.676	0.952
**50-99**	0.130	0.130	0.130	0.348	1.449	1.449	2.00
HIV mortality rates (per year) in the absence of ART, μH							
**>500**	0.312	0.039	0.039	0.005	0.004	0.005	0.005
**350-500**	0.382	0.048	0.048	0.011	0.01	0.013	0.013
**250-349**	0.466	0.058	0.058	0.026	0.026	0.036	0.032
**200-249**	0.466	0.058	0.058	0.061	0.069	0.096	0.08
**100-199**	0.569	0.071	0.071	0.139	0.185	0.258	0.203
**50-99**	0.569	0.071	0.071	0.321	0.499	0.691	0.513
**<50**	0.569	0.071	0.071	0.737	1.342	1.851	1.295

Web Table 4. ART mortality parameters, μA, male (per year)

	Age Group, years						
**CD4**	**0-4**	**5-9**	**10-14**	**15-24**	**25-34**	**35-44**	**45+**
0-6 months on ART							
**>500**	0.0568	0.0071	0.0067	0.0050	0.0035	0.0050	0.0050
**350-500**	0.2567	0.0321	0.0301	0.0115	0.0095	0.0134	0.0126
**250-349**	0.1945	0.0241	0.0228	0.0264	0.0256	0.0359	0.0319
**200-249**	0.1945	0.0241	0.0228	0.0607	0.0563	0.0587	0.0594
**100-199**	0.6814	0.0852	0.0799	0.1113	0.0929	0.0980	0.1039
**50-99**	0.6814	0.0852	0.0799	0.1810	0.1525	0.1619	0.1762
**<50**	0.6814	0.0852	0.0799	0.3974	0.3373	0.3605	0.4009
7-12 months on ART							
**>500**	0.1501	0.0188	0.0088	0.0050	0.0035	0.0050	0.0050
**350-500**	0.2148	0.0269	0.0125	0.0115	0.0095	0.0134	0.0126
**250-349**	0.2039	0.0269	0.0119	0.0244	0.0256	0.0323	0.0319
**200-249**	0.2039	0.0269	0.0119	0.0258	0.0332	0.0341	0.0451
**100-199**	0.3850	0.0481	0.0225	0.0323	0.0417	0.0433	0.0584
**50-99**	0.3850	0.0481	0.0225	0.0405	0.0523	0.0548	0.0751
**<50**	0.3850	0.0481	0.0225	0.0583	0.0753	0.0797	0.1112
>12 months on ART							
**>500**	0.0636	0.0080	0.0037	0.0050	0.0035	0.0050	0.0050
**350-500**	0.0910	0.0114	0.0053	0.0086	0.0095	0.0101	0.0102
**250-349**	0.0864	0.0108	0.0050	0.0092	0.0117	0.0109	0.0114
**200-249**	0.0864	0.0108	0.0050	0.0098	0.0125	0.0118	0.0127
**100-199**	0.1632	0.0204	0.0095	0.0129	0.0165	0.0161	0.0190
**50-99**	0.1632	0.0204	0.0095	0.0168	0.0216	0.0216	0.0268
**<50**	0.1632	0.0204	0.0095	0.0251	0.0333	0.0333	0.0438

Web Table 5. ART mortality parameters, μA, female (per year)

	Age Group, years						
**CD4**	**0-4**	**5-9**	**10-14**	**15-24**	**25-34**	**35-44**	**45+**
0-6 months on ART							
**>500**	0.0680	0.0085	0.0080	0.0050	0.0035	0.0050	0.0050
**350-500**	0.3074	0.0384	0.0360	0.0115	0.0095	0.0134	0.0126
**250-349**	0.2329	0.0291	0.0273	0.0264	0.0256	0.0359	0.0319
**200-249**	0.2329	0.0291	0.0273	0.0529	0.0431	0.0445	0.0433
**100-199**	0.8160	0.1020	0.0957	0.0866	0.0719	0.0754	0.0783
**50-99**	0.8160	0.1020	0.0957	0.1415	0.1187	0.0126	0.1352
**<50**	0.8160	0.1020	0.0957	0.3118	0.2641	0.2819	0.3120
7-12 months on ART							
**>500**	0.1430	0.0179	0.0083	0.0050	0.0035	0.0050	0.0050
**350-500**	0.2046	0.0256	0.0119	0.0115	0.0095	0.0134	0.0126
**250-349**	0.1942	0.0243	0.0113	0.0183	0.0235	0.0237	0.0299
**200-249**	0.1942	0.0243	0.0113	0.0193	0.0248	0.0251	0.0320
**100-199**	0.3667	0.0458	0.0214	0.0245	0.0315	0.0323	0.0425
**50-99**	0.3667	0.0458	0.0214	0.0309	0.0399	0.0414	0.0556
**<50**	0.3667	0.0458	0.0214	0.0449	0.0580	0.0610	0.0840
>12 months on ART							
**>500**	0.0606	0.0076	0.0035	0.0050	0.0035	0.0050	0.0042
**350-500**	0.0867	0.0108	0.0051	0.0058	0.0074	0.0062	0.0046
**250-349**	0.0823	0.0103	0.0050	0.0063	0.0080	0.0069	0.0055
**200-249**	0.0823	0.0103	0.0048	0.0068	0.0086	0.0076	0.0065
**100-199**	0.1554	0.0194	0.0091	0.0092	0.0118	0.0110	0.0115
**50-99**	0.1554	0.0194	0.0091	0.0123	0.0157	0.0152	0.0176
**<50**	0.1554	0.0194	0.0091	0.0188	0.0242	0.0244	0.0310

1.3 Demographic model

The model is age structured in 5-year age bins (0-4, 5-9, ... 75-80) and a single bin representing those 80+ (17 age groups in total). Demographic parameters are taken from the UN population Division [18].

Births are modelled using the crude birth rate (/1000 population) and are added into the susceptible population (age 0-4) as a fixed event at the start of each year. Aging is also modelled as a discrete event following the method of Schenzle [19].

Migration, m_i is modelled as a continuous process based on estimated net numbers of migrants by age, i, and calendar year. Migration does not depend on disease state (TB or HIV). Instead, the total number of migrants in a given age group is divided between disease states based on the relative size of compartments.

Deaths are modelled using mortality rates derived from UN population division life tables. Because these mortality rates include deaths due to HIV and TB it is necessary to correct for disease induced mortality in the model to derive the age specific background mortality rate, μ_i. This is achieved by reducing the UN estimated mortality rates at each time point by the rate of disease (HIV and TB) mortality. In the same way, the HIV mortality rates (see section 1.2) include deaths in HIV-infected individuals due to TB and must also be corrected to avoid double counting of these deaths in the model. This correction is applied up to 2015 after which the reduction in background mortality is fixed at the 2015 levels. This allows changes in future TB burden to be reflected in the overall mortality. Web Figure 2 shows the comparison of the model population to UN population estimates.

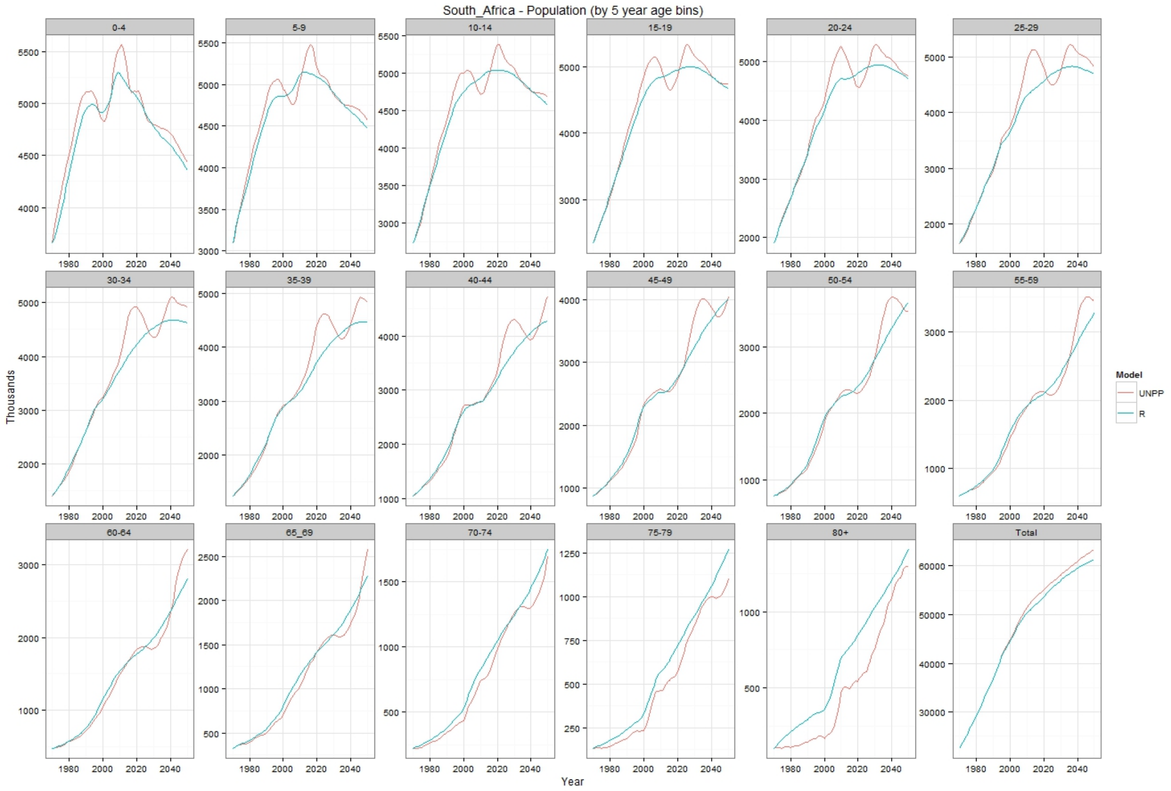

Web Figure 2. Comparison of demographic projections

1.4 Base-case care and control assumptions

The following sections describe the model assumptions regarding the screening, diagnosis and treatment of TB cases in the baseline model. In the base-case, we assume that all activities continue at their current levels into the future. In a secondary analysis we also included certain activities proposed in the National Strategic Plan for HIV and TB [20]. These are described in section 1.6.

Screening

The rates of screening (r_s) are largely unknown (particularly for passive presentation).

We assume that the rates of passive screening (of those with or without TB) are independent of HIV status but do depend on TB status. Rates of passive screening are also assumed to be lower for individuals with smear-negative TB (by a factor of 0.8) compared to those with smear-positive TB. The rates of passive screening (in those with and without TB) are estimated by fitting the model to incidence, notifications and testing data (see Web Appendix 2). Passive screening is assumed to be based on presence of any symptom with a sensitivity and specificity of 84 (95% CI: 76–93) and 74 (95% CI: 53–95) respectively [21].

We assume there is no intensified screening for TB in HIV-uninfected individuals or those with unknown status pre-2010. Post 2010 values are estimated by fitting the model to case-finding data reported to the NDOH (see Web Appendix 2). In the baseline, ICF in this population is assumed to be based on presence of prolonged cough (>2 weeks). This assumption is based on province level case-finding data where the ratio of symptomatic to screened was approximately 7%, consistent with the higher specificity of prolonged cough compared to any symptom (see Web Table 6). In addition, the screening tool used (see Web Figure 3) only requests sputum collection among HIV-uninfected individuals if prolonged cough is present. We assumed a sensitivity and specificity of 35 (95% CI: 24–46) and 95 (95% CI: 93-97) respectively for cough screening [21].

Web Table 6. Data from TB control and management performance monitoring (2014). Only includes those provinces for which screening numbers were reported (Eastern Cape, Free State, Limpopo, Northern Cape, Western Cape).

% of Head Count Screened	% of Those Screened With Symptoms	% of Those With Symptoms Tested
34.7	7.6	92.7

Active screening in HIV+ individuals (with known status) occurs as part of HIV care. We assume that individuals in HIV care make on average 6 clinic visits per year. According to the WHO notifications database, approximately 40% of those enrolled in care were asked about TB symptoms at their last visit [22]. While there is likely to be considerable uncertainty in this value it does allow us to estimate an approximate rate at which those in HIV care are screened for TB of 2.4 /year.

Web Figure 3. Screening tool taken from South African National TB Management Guidelines (2014) [23].

ANNEXURE 7:
TB SCREENING TOOL

TB SYMPTOM SCREENING TOOL FOR ADULTS AND CHILDREN

PATIENT DETAILS

Surname:	First Name:
Physical Address:	Age:
Telephone Number:	Patient folder Number:

MEDICAL HISTORY

Close contact of a person with infectious TB:	Yes	No	Unknown	(Tick √)
Type of index patient:	DS-TB	Rif Resistant TB	MDR-TB or XDR-TB	
Diabetic:	Yes	No	Unknown	
HIV Status:	Positive	Negative	Unknown	
Other: (Specify)				

TB SYMPTOM SCREEN

1. ADULTS

**Symptoms** (Tick √)	Yes	No
Cough of 2 weeks or more OR of any duration if HIV positive		
Persistent fever of more than two weeks		
Unexplained weight loss >1.5kg in a month		
Drenching night sweats		

2. CHILDREN

**Symptoms** (Tick √)	Yes	No
Cough of 2 weeks or more which is not improving on treatment		
Persistent fever of more than two weeks		
Documented weight loss/ failure to thrive (check Road to Health Card)		
Fatigue (less playful/ always tired)		

If "Yes" to one or more of these questions, consider TB.
If the patient is coughing, collect sputum specimen and send it for Xpert testing.
If the patient is not coughing but has the other symptoms, clinically assess the patient or refer for further investigation.

Date of last TB test:		
Patient referred for assessment and investigation:	Yes	No
Date of referral:	Facility name:	
Name:	Date:	

114 National Tuberculosis Management Guidelines 2014

Diagnostic algorithm

Of those with possible TB identified via screening we assume 90% provide a sputum sample for testing (see Web Table 6).

These individuals will enter one of 2 diagnostic pathways defined by the initial test: smear microscopy (SSM) or Gene Xpert (GX). Web Figure 4 shows a simplified representation of the algorithm based on the SA guidelines [23]. In each case, some proportion of the population will receive smear or Xpert as initial test. This proportion is assumed to vary over time to capture historical changes in Xpert utilization (see Web Table 7). Of those who test negative, some proportion will receive follow up investigation. This proportion will vary over time and depends on HIV status and type of initial test (see below).

Web Figure 4. Simplified representation of the SA diagnostic algorithm. SSM = sputum smear microscopy. GX = Gene Xpert.

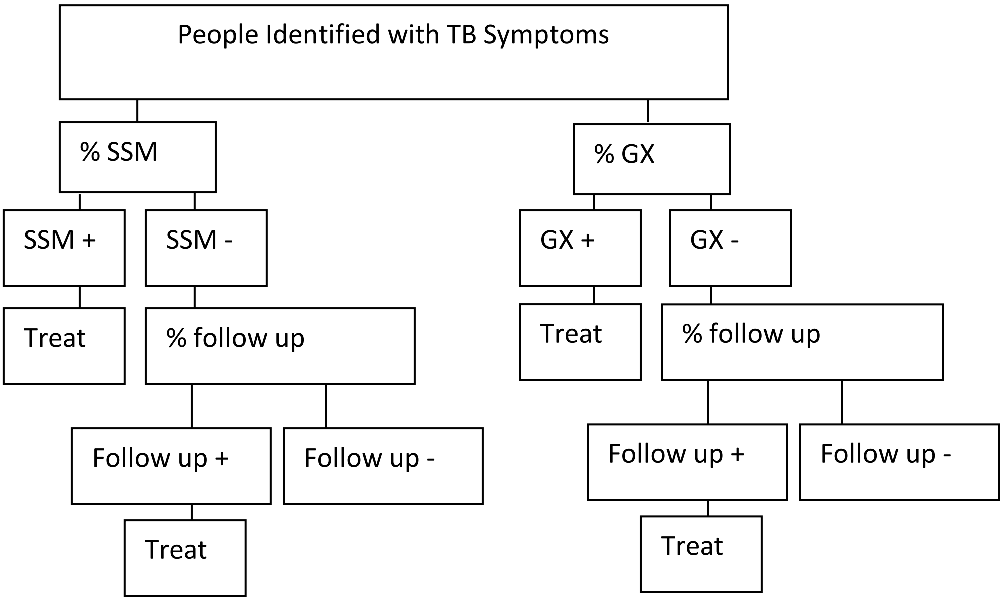

<u>Smear-negative algorithm (2009 Guidelines)</u>

Following a negative smear, HIV-uninfected individuals should be treated with broad spectrum antibiotics. If no improvement is observed after these antibiotics TB should be suspected and further smear and/or x-ray performed. HIV-infected individuals should also be started on antibiotics but with immediate third smear and/or x-ray.

<u>Xpert-positive algorithm (2014 Guidelines)</u>

Following a negative Xpert, HIV-uninfected individuals should be started on broad spectrum antibiotics and referred for further investigation if no improvement after 1 week. HIV-infected

individuals should be assessed clinically, given x-ray (if available) and have sputum taken for culture/DST.

From the Xtend study [24] we calculated the percentage of individuals with an initial negative test who are followed up (by initial test type and HIV status) (see Web Table 8). The majority of those with evidence of follow-up had culture results (85%).

In addition, some TB cases may be diagnosed on clinical grounds. In the model, this is captured by allowing some proportion of those with a negative test (who do not receive culture) to be investigated clinically. The proportion investigated clinically was adjusted to try to match the proportion of notified TB cases in South Africa without bacteriological confirmation. The proportion of cases without laboratory confirmation (i.e. clinical diagnosis) reported to WHO in 2016 was 33%. The modelled estimates ranged from 12% to 39%.

The assumed sensitivity and specificity of the different steps in the diagnostic algorithm are given in Web Table 9.

Web Table 7. Utilization of Xpert as the first-line diagnostic test

Year	Utilization of Xpert as First-Line Diagnostic Test, %
2010	0
2011	0
2013	65
2016	80

Web Table 8. Percentage of those with initial test negative with evidence of follow up (by HIV status and type of initial test)

	Microscopy			Xpert		
**HIV status**	+	Unknown	−	+	Unknown	−
**% followed up**	32.5	18.4	5.5	13.8	5.0	8.6

Web Table 9. Sensitivity and specificity of diagnostic tools used in the model

Test	Sensitivity		Specificity
	Smear +	Smear -	
Smear	1	0	97.4 (96.5-98.2)
Xpert	98.2 (96.9-99.1)	72.5 (65.5-78.8)	99.2 (98.2-99.7)
Culture	1	1	1
Clinical diagnosis	0.24 (0.1-0.51)	0.24 (0.1-0.51)	0.95 (0.92-0.97)

Initial loss to follow-up (ILTFU)

Information on ILTFU, the gap between positive diagnosis and treatment initiation is limited. Based on a systematic review published in 2014 [25] and data from the Xtend study [26] we assumed that 17% of those with a positive diagnosis did not initiate treatment. This is parameterised in the model as the proportion of those diagnosed with TB who initiate treatment, κ_S = 100-17 = 83%.

For drug-resistant cases, loss to follow up is based on the proportion of lab confirmed MDR cases reported to have started treatment within 1 year (see Web Table 10).

Web Table 10. Linkage of lab-confirmed MDR cases to treatment

Year	Proportion of Lab-Confirmed MDR Cases Initiated on Treatment Within 1 Year
2007	44.88
2008	49.17
2009	45.68
2010	71.93
2011	55.95
2012	45.86

Treatment success, τ

For simplicity, individuals who start treatment are divided into those who are successfully treated (completed + cured) and those who are not. Treatment success was assumed to be independent of HIV status but does depend on drug resistance. Values of treatment success for drug-susceptible cases are shown in table A11 and are assumed to remain at current levels in the future. MDR treatment success, τ_M, was assumed to be between 48 and 52% [27].

Web Table 11. Treatment success for DS-TB cases, τ_S (WHO TB database [27])

Year	Treatment Success (Completed + Cured), %
2006	70.0
2007	68.8
2008	71.3
2009	72.3
2010	70.8
2011	75.4
2012	76.1
2013	77.9
2014	77.5

1.5 NTP Scenario

In a secondary analysis, we adapted the base case to include the following activities planned in South Africa as part of the National Strategic Plan 2017-2022 [20]: reducing pre-treatment loss to follow up by 80% (from 17% to 4%) by 2021 via the introduction of SMS reminders and mobile outreach teams; the introduction of short-course MDR treatment [28] alongside continued use of bedaquiline for pre-XDR and XDR TB [29].

It has been estimated that 70% of patients will be eligible for the shortened MDR regimen, 10% for a bedaquiline regimen and 20% for conventional MDR treatment resulting in potential savings in costs and nurse time. However, given the low quality of evidence for the effectiveness of the shortened regimen [28] we assumed that its introduction would not improve overall treatment outcomes.

1.6 Knowledge of HIV status

Screening and follow-up investigations in initial test negatives depend on known HIV status. Knowledge of HIV status is not included in the model. Instead the true HIV positive population is divided into those who are on ART and those who are not on ART.

The 2012 South African HIV survey [30] reports on the proportion of individuals (by sex and true status) who reported knowing their HIV status (Web Table 12).

Web Table 12. Proportion of individuals (>15) who knew their HIV status (by sex and true HIV status)

HIV Status	Male		Female	
	HIV-	HIV+	HIV-	HIV+
**Known**	35.6	37.8	45.0	55.0
**Unknown**	64.4	62.2	55.0	45.0

The survey also found that approximately 60% of HIV is in females. Ignoring the fact that approximately 3% of HIV is estimated to be in children under 15, we can estimate that (0.6*55 + 0.4*37.8) = 48% of true HIV positives knew their status in 2012. The survey also reports that 25.7% and 34.7% of males and females living with HIV were on ART giving an overall coverage of 31.1% (assuming as before that 60% of HIV is in females).

According to UNAIDS 2016 estimates [31], there were 7000000 (6700000-7400000) people living with HIV in South Africa and 3384160 (48% (46-52)) were on ART in 2015. The UNAIDS gap report suggests that in sub-Saharan Africa approximately 90% of HIV positive individuals who know their HIV status are receiving ART. From this we can estimate that 53% of individuals knew their status in 2015.

The percentage of the HIV positive population not on ART who know their status can then be derived using the following:

$$b = a + c(1 - a),$$

where a is the percentage of the HIV-infected population on ART, b is the percentage of all PLWHIV who know their status and c is the percentage of those not on ART who know their status.

Web Table 13. Calculated knowledge of HIV status among those not on ART

Year	a) % Living with HIV on ART	b) % Who Know Status	c) % Not on ART Who Know Status
**2012**	31	48	24.6
**2015**	48	53	9.6

1.7 Model equations

The model is implemented as a set of differential equations in the C programming language as a compiled DLL and solved using the deSolve package in R.

The following equations describe the progression for HIV-uninfected individuals. As mentioned above, aging and births are modelled as a fixed event at the start of each year. Subscript *i* indicates age.

HIV-uninfected persons

$$\frac{dS_i}{dt} = -(\lambda_S+\lambda_M)S_i - h_iS_i - \mu_iS_i + \frac{m_iS_i}{N_i}$$

$$\frac{dLsn_i}{dt} = \lambda_S(1-a_i)(S_i + (1-p)(FTn_i + (1-g)Lmn_i)) - (v_i + \lambda_S a_i(1-p))Lsn_i - \lambda_M(1-p)(a_iLsn_i + (1-a_i)gLsn_i) + r_IIsn_i + r_NNsn_i - \delta\kappa_S\tau_SLsn_i - h_iLsn_i - \mu_iLsn_i + \frac{m_iLsn_i}{N_i}$$

$$\frac{dLsp_i}{dt} = \lambda_S(1-a_i)(1-p)(FTp_i + (1-g)Lmp_i) - \big(v_i + \lambda_S a_i(1-p)\big)Lsp_i - \lambda_M(1-p)(a_iLsp_i + (1-a_i)gLsp_i) + r_IIsp_i + r_NNsp_i - \delta_F\kappa_S\tau_SLsp_i + \delta_T\kappa_S\tau_S(Nsn_i + Nsp_i + Isn_i + Isp_i) - h_iLsp_i - \mu_iLsp_i + \frac{m_iLsp_i}{N_i}$$

$$\frac{dLmn_i}{dt} = \lambda_M(1-a_i)(S_i + (1-p)g(FTn_i + Lsn_i)) - (v_i + \lambda_M a_i(1-p))Lmn_i - \lambda_S(1-p)(a_iLmn_i + (1-a_i)(1-g)Lmn_i) + r_IImn_i + r_NNmn_i - h_iLmn_i - \mu_iLmn_i + \frac{m_iLmn_i}{N_i}$$

$$\frac{dLmp_i}{dt} = \lambda_M(1-a_i)(1-p)g(FTp_i + Lsp_i) - \big(v_i + \lambda_M a_i(1-p)\big)Lmp_i - \lambda_S a_i(1-p)Lmp_i - \lambda_S(1-a_i)(1-p)(1-g)Lmp_i + r_IImp_i + r_NNmp_i + \delta_T\kappa_M\tau_M(Nmn_i + Nmp_i + Imn_i + Imp_i) - h_iLmp_i - \mu_iLmp_i + \frac{m_iLmp_i}{N_i}$$

$$\frac{dNsn_i}{dt} = \lambda_S a_i(1-\sigma_i)\big(S_i + (1-p)(Lsn_i + Lmn_i + FTn_i)\big) + v_i(1-\sigma_i)Lsn_i - (\vartheta + r_N + \mu N_i + \mu_i + h_i)Nsn_i - \frac{m_i Nsn_i}{N_i} - \delta_T(1-\kappa_S)Nsn_i$$

$$\frac{dNsp_i}{dt} = \lambda_S a_i(1-\sigma_i)(1-p)(Lsp_i + Lmp_i + FTp_i) + v_i(1-\sigma_i)Lsp_i - (\vartheta + r_N + \mu N_i + \mu_i + h_i)Nsp_i - \frac{m_i Nsp_i}{N_i} - \delta_T(1-\kappa_S)Nsp_i + \delta_T\kappa_S\tau_S(Nsn_i + Nsp_i)$$

$$\frac{dIsn_i}{dt} = \lambda_S a_i\sigma_i\big(S_i + (1-p)(Lsn_i + Lmn_i + FTn_i)\big) + v_i\sigma_i Lsn_i + \vartheta Nsn_i - (r_I + \mu I_i + \mu_i + h_i)Isn_i - \frac{m_i Isn_i}{N_i} - \delta_T(1-\kappa_S)Isn_i$$

$$\frac{dIsp_i}{dt} = \lambda_S a_i\sigma_i(1-p)(Lsp_i + Lmp_i + FTp_i) + v_i\sigma_i Lsp_i + \vartheta Nsp_i - (r_I + \mu I_i + \mu_i + h_i)Isp_i - \frac{m_i Isp_i}{N_i} - \delta_T(1-\kappa_S)Isp_i + \delta_T\kappa_S\tau_S(Isn_i + Isp_i)$$

$$\frac{dNmn_i}{dt} = \lambda_M a_i(1-\sigma_i)\big(S_i + (1-p)(Lsn_i + Lmn_i + FTn_i)\big) + v_i(1-\sigma_i)Lmn_i - (\vartheta + r_N + \mu N_i + \mu_i + h_i)Nmn_i - \frac{m_i Nmn_i}{N_i} - \delta_T(1-\kappa_M)Nmn_i$$

$$\frac{dNmp_i}{dt} = \lambda_M a_i(1-\sigma_i)(1-p)(Lsp_i + Lmp_i + FTp_i) + v_i(1-\sigma_i)Lmp_i - (\vartheta + r_N + \mu N_i + \mu_i + h_i)Nmp_i - \frac{m_i Nmp_i}{N_i} - \delta_T(1-\kappa_M)Nmp_i + \delta_T\kappa_M\tau_M(Nmn_i + Nmp_i)$$

$$\frac{dImn_i}{dt} = \lambda_M a_i\sigma_i\big(S_i + (1-p)(Lsn_i + Lmn_i + FTn_i)\big) + v_i\sigma_i Lmn_i + \vartheta Nmn_i - (r_I + \mu I_i + \mu_i + h_i)Imn_i - \frac{m_i Imn_i}{N_i} - \delta_T(1-\kappa_M)Imn_i$$

$$\frac{dImp_i}{dt} = \lambda_M a_i\sigma_i(1-p)(Lsp_i + Lmp_i + FTp_i) + v_i\sigma_i Lmp_i + \vartheta Nmp_i - (r_I + \mu I_i + \mu_i + h_i)Imp_i - \frac{m_i Imp_i}{N_i} - \delta_T(1-\kappa_M)Imp_i + \delta_T\kappa_M\tau_M(Imn_i + Imp_i)$$

$$\frac{dFTn_i}{dt} = \delta\kappa_S\tau_S Lsn_i - \lambda_S(1-p)FTn_i - \lambda_M(1-p)(a_i + (1-a_i)g)FTn_i - h_i FTn_i - \mu_i FTn_i + \frac{m_i FTn_i}{N_i}$$

$$\frac{dFTp_i}{dt} = \delta\kappa_S\tau_S Lsp_i - \lambda_S(1-p)FTp_i - \lambda_M(1-p)(a_i + (1-a_i)g)FTp_i - h_i FTp_i - \mu_i FTp_i + \frac{m_i FTp_i}{N_i}$$

HIV-infected persons not on ART

The equations for HIV-infected individuals not on ART are similar to those described above, but with additional terms describing acquisition of HIV (h_i), progression through CD4 states $\varepsilon_{i,j}$, ART initiation ($ART_{i,j}$) and HIV associated mortality ($\mu H_{i,j}$) as illustrated in the equation for the susceptible population below. In addition, HIV specific parameters are used as described previously.

$$\frac{dSH_{i,j}}{dt} = -(\lambda_S+\lambda_M)SH_{i,j} + h_i\gamma_{i,j}S_i - \varepsilon_{i,j}SH_{i,j} + \varepsilon_{i,j-1}SH_{i,j-1} - (\mu_i + \mu H_{i,j} + ART_{i,j})SH_{i,j} + m_iSH_{i,j}/N_i$$

HIV-infected persons on ART

The equations for HIV-infected individuals on ART include additional terms describing initiation of ART ($ART_{i,j}$), progression through time on ART ($\eta_{i,j,l}$) HIV associated mortality while on ART ($\mu A_{i,j,l}$) as illustrated in the equation for the susceptible population below. In addition, ART adjusted parameters are used as described previously.

$$\frac{dSA_{i,j,l}}{dt} = -(\lambda_S+\lambda_M)SA_{i,j,l} - \eta_{i,j,l}SA_{i,jl} + \eta_{i,j,l-1}SA_{i,j,l-1} - (\mu_i + \mu_{A_{i,j,l}})SA_{i,j,l} + ART_{i,j}SH_{i,j} + m_iSA_{i,j,l}/N_i$$

Force of infection

$$\lambda_S = \beta(\textstyle\sum_i(Isn_i + Isp_i) + \sum_{i,j}\left(IsnH_{i,j} + IspH_{i,j}\right) + \sum_{i,j,l}\left(IsnA_{i,j,l} + IspA_{i,j,l}\right) + \omega(\sum_i(Nsn_i + Nsp_i) + \sum_{i,j}\left(NsnH_{i,j} + NspH_{i,j}\right) + \sum_{i,j,l}(NsnA_{i,j,l} + NspA_{i,j,l})))/N$$

$$\lambda_M = f\beta(\textstyle\sum_i(Imn_i + Imp_i) + \sum_{i,j}\left(ImnH_{i,j} + ImpH_{i,j}\right) + \sum_{i,j,l}\left(ImnA_{i,j,l} + ImpA_{i,j,l}\right) + \omega(\sum_i(Nmn_i + Nmp_i) + \sum_{i,j}\left(NmnH_{i,j} + NmpH_{i,j}\right) + \sum_{i,j,l}(NmnA_{i,j,l} + NmpA_{i,j,l})))/N$$

Screening and diagnosis

For each active disease state, the rate of true positive diagnosis is given by:

$$\delta_T = r_s se_s p_t(se_t + (1 - se_t)p_f se_f + (1 - se_t)\left(1 - p_f\right)p_c se_c)$$

For each non-active disease state, the rate of false positive diagnosis is:

$$\delta_F = r_s(1 - sp_s)p_t((1 - sp_t) + sp_t p_f\left(1 - sp_f\right) + sp_t\left(1 - p_f\right)p_c(1 - sp_c))$$

Web Table 14 defines the parameters used in these expressions. Values for these parameters (by disease state and HIV status) are discussed in section 1.4.

Web Table 14. Definitions of parameters in the diagnostic rate expressions

Parameter	Description
r_s	Rate at which individuals are screened
se_s	Sensitivity of screening
sp_s	Specificity of screening
p_t	Proportion of those screening positive who provide a sputum sample for testing
se_t	Sensitivity of initial testing
sp_t	Specificity of initial testing
p_f	Proportion of those testing negative who are followed up as per guidelines
se_f	Sensitivity of follow-up testing
sp_f	Specificity of follow-up testing
p_c	Proportion of those not followed up who are clinically assessed
se_c	Sensitivity of clinical diagnosis
sp_c	Specificity of clinical diagnosis

WEB APPENDIX 2

Model Calibration

To avoid issues with trying to identify multiple correlated parameters, the model was calibrated in a two-step process by varying selected model parameters to minimise the weighted sum of square differences between the model and the observed data. Fitting was carried out using a Nelder-Mead algorithm via the FME package in R.

Web Table 15 lists the calibration data and sources used.

The process consisted of the following steps.

1. Sample values for all uncertain model parameters (including natural history and programmatic parameters).

2. Fit the model to the 1990 and 1991 HIV negative TB incidence and TB mortality rates and overall TB notification rates (/100,000) by varying the following:
    a. contact rate, β
    b. baseline passive screening rate, *kbase*

3. Using these fitted values, fit the model to:
    a. TB incidence and TB mortality rates by HIV status (/100,000) (1990-2015)
    b. total TB notification rates (/100,000) (1990-2015)
    c. MDR TB treatment initiations (/100,000) (2007-2015)
    d. Population tested for TB (/100,000) from 2004-2012
    e. Population screened for TB in 2014 and 1015

    by varying the following:

    a. increase in passive screening rate in HIV negatives, *kincrease*
    b. the rate of acquisition of MDR, *e*
    c. scaling factor that modifies the protection against TB disease and TB death when on ART, *ART_mult*
    d. scaling factor that modifies the mortality rate from TB in HIV-infected individuals, *HIV_mult*
    e. the rate of ICF in the general population (HIV-uninfected and unknown status)

4. Repeat steps 1–3 to generate *N* = 1000 fitted parameter sets.

Web Table 15. **Calibration data**. Notifications, incidence and mortality were taken from the WHO notifications [22] and burden databases [32]. Screening numbers were taken from SA NDOH reports and are assumed to represent the total number of screens conducted (passive plus active (ICF)). Test data is based on [33]

	**Notifications (number)**		**Incidence (/100k)**		**Mortality (/100k)**		**Screened (millions)**	**Tested (/100k)**
**Year**	**All**	**MDR**	**HIV-**	**HIV+**	**HIV-**	**HIV+**	**All**	**All**
1990	80400	-	299	14	47	1	-	-
1991	77652	-	281	28	46	2	-	-
1992	82539	-	251	48	46	3	-	-
1993	89786	-	219	71	45	6	-	-
1994	90292	-	194	95	44	9	-	-
1995	73917	-	181	121	47	13	-	-
1996	109328	-	181	152	51	21	-	-
1997	125913	-	189	190	54	33	-	-
1998	142281	-	206	233	59	51	-	-
1999	148164	-	229	280	64	75	-	-
2000	151239	-	253	332	68	102	-	-
2001	148257	-	282	384	73	130	-	-
2002	215120	-	310	436	74	158	-	-
2003	227320	-	336	484	76	181	-	-
2004	267290	-	359	524	75	195	-	2353
2005	270178	-	377	555	75	197	-	2773
2006	303114	-	387	576	73	190	-	3347
2007	315315	3334	391	586	69	178	-	3559
2008	348241	4031	389	588	65	170	-	4376
2009	360183	4143	384	583	62	167	-	4559
2010	354786	5313	377	571	59	163	-	6160
2011	362453	5643	319	603	55	149	-	7164
2012	323664	6494	322	570	51	130	-	6415
2013	312380	8439	330	530	47	129	-	-
2014	306166	11708	325	509	44	134	28	-
2015	-	-	-	-	-	-	36	-

WEB APPENDIX 3

Additional Results—Secondary Analysis

The secondary analysis considered a revised the base-case including the following activities planned in South Africa as part of the National Strategic Plan: reducing PLTFU by 80% (from 17% to 4%) by 2021; the introduction of short-course MDR treatment alongside continued use of bedaquiline for pre-XDR and XDR TB. This analysis allowed us to explore how the impact and resource use of the ICF strategies may be altered by other improvements in the TB program.

These additional activities resulted in a greater decline in incidence in the base-case and in each intervention scenario. However, the incremental benefits of each intervention were slightly reduced as the effects were not additive when combined with the improved base-case.

Web Figure 5 compares the additional reductions in incidence due to each intervention in the primary and secondary analysis.

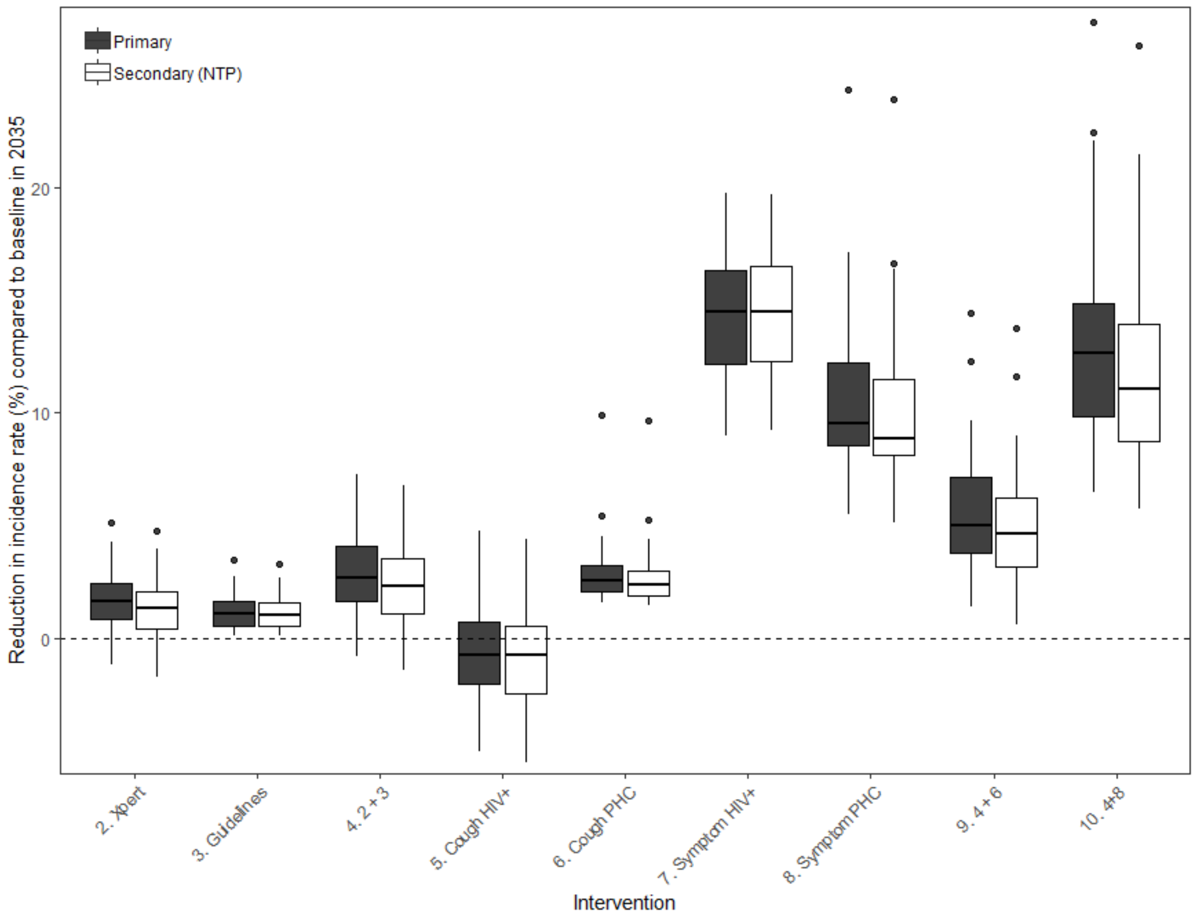

Web Figure 5. Impact on incidence. Percentage reduction in incidence rate in 2035 compared to the baseline (intervention 1). Shading indicates the base-case assumption. Boxes show the 25th–75th percentile range, whiskers indicate 1.5 times the interquartile range and black circles show outliers.

The total resource use in the secondary analysis was also slightly lower than in the primary analysis presented in the main text (Web Figure 6). However, the differences in resource use were small and did not change our qualitative conclusions that resource constraints may limit the impact of case-finding interventions.

Web Figure 7 shows the impact of each intervention with and without constraints in the NTP base-case. The only significant difference between these results and those of the primary analysis is that scale up of cough based screening among PHC clinic attendees (Intervention 6) was not affected by the HR constraints in the secondary analysis. However, the impact of interventions involving symptom screening (interventions 7,8 and 10; the most effective interventions in the unconstrained scenario) was still significantly reduced by the HR constraints.

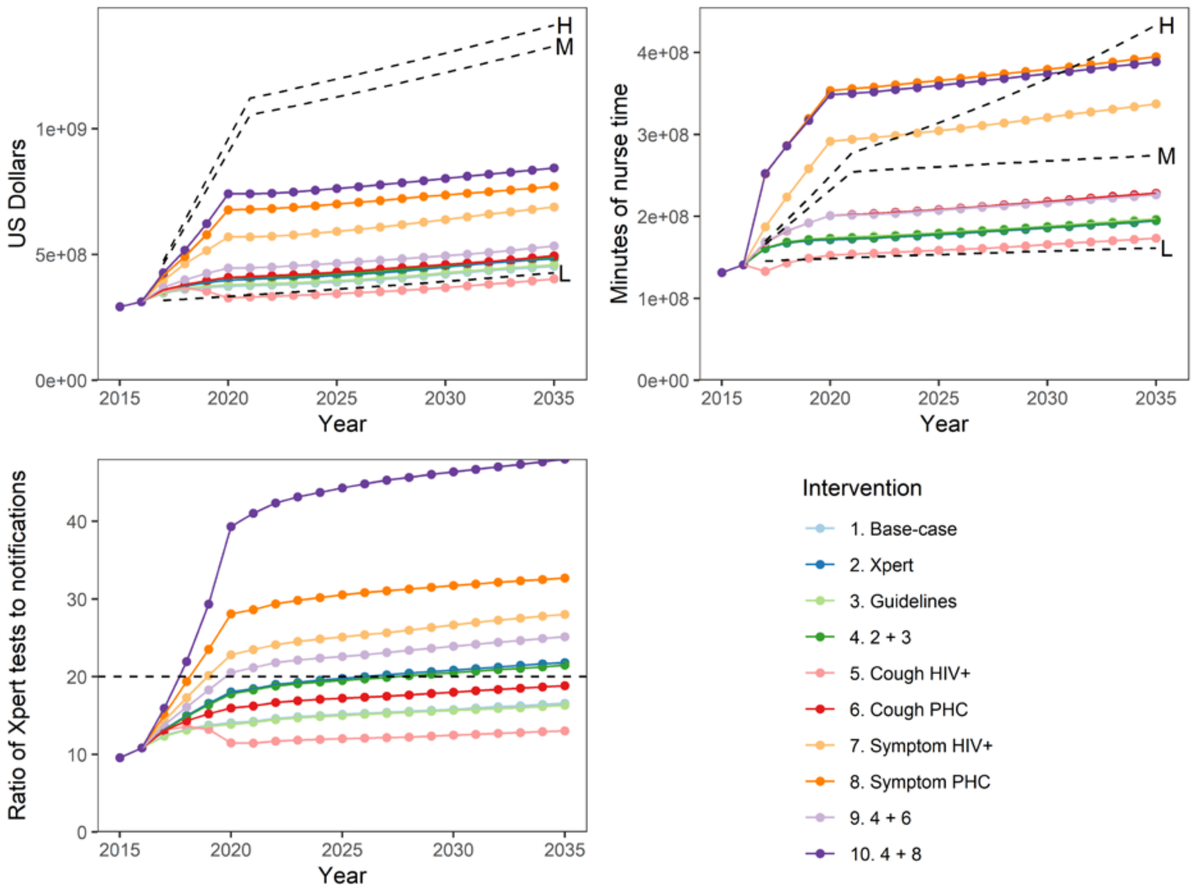

Web Figure 6. **Projection of future costs, HR requirements and Xpert to Notification ratio in the secondary analysis**. Colored lines show the median model prediction for each intervention from 2015 to 2035. Top right: Total costs of TB control activities (in US dollars). Top right: Nurse time spent on TB activities (in minutes). Bottom left: Number of Xpert tests per TB notification. Dashed black lines show the low (L), medium (M) and high (H) constraints for total cost and nurse minutes. In the Xpert panel (bottom left) only the single constraint (a ratio of 20:1) is shown.

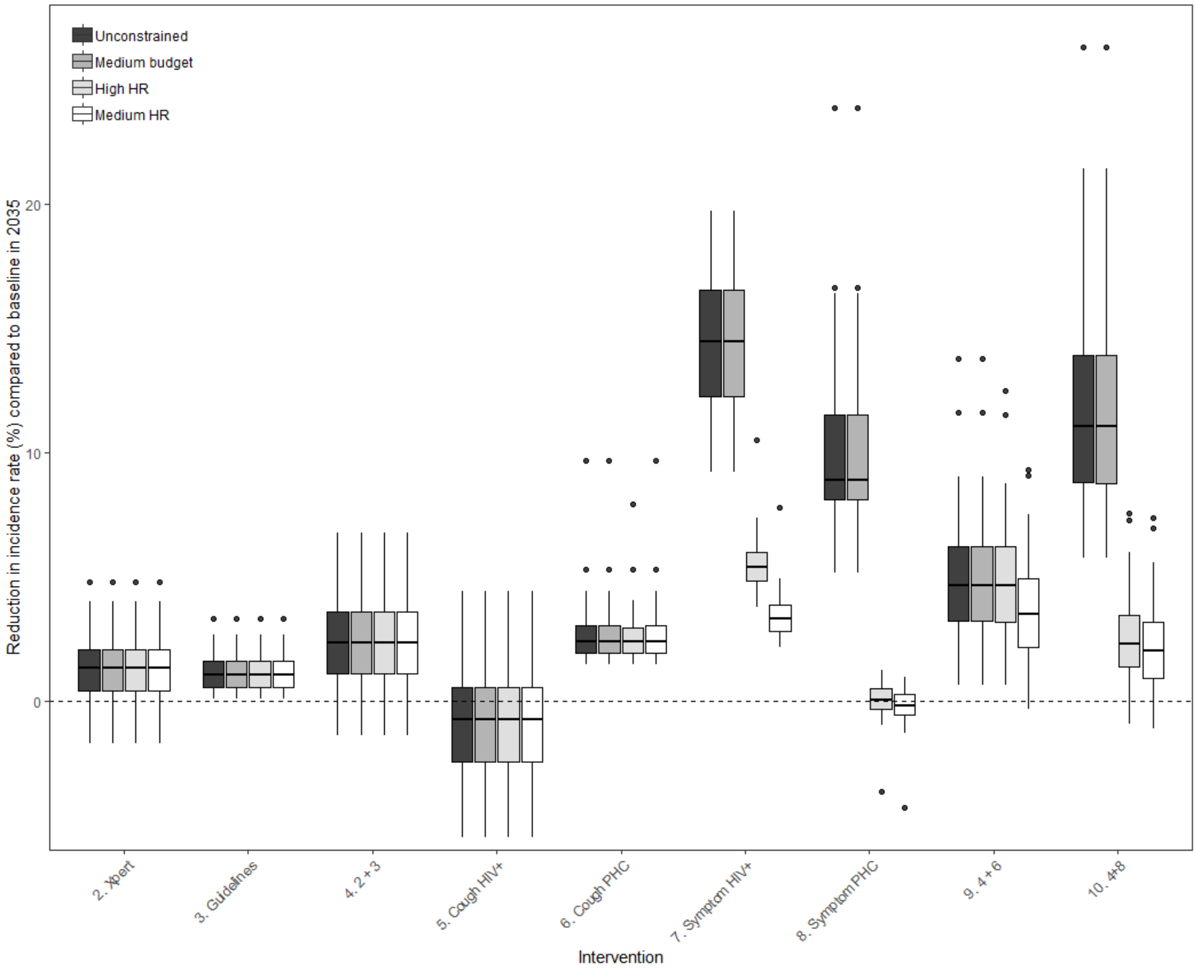

Web Figure 7. Impact on incidence—secondary analysis. Percentage reduction in incidence rate in 2035 compared to the baseline (intervention 1). Shading indicates the constraints applied to the model. Boxes show the 25th–75th percentile range, whiskers indicate 1.5 times the interquartile range and black circles show outliers. The high budget constraint is not shown as results are the same as for the medium budget constraint. Values above 0 (dashed horizontal line) indicate a larger reduction in incidence compared to the baseline.

Web References

1. Houben, R.M., et al., *TIME Impact - a new user-friendly tuberculosis (TB) model to inform TB policy decisions.* BMC Med, 2016. **14**.
2. Menzies, N.A., et al., *Population health impact and cost-effectiveness of tuberculosis diagnosis with Xpert MTB/RIF: a dynamic simulation and economic evaluation.* PLoS Med, 2012. **9**(11).
3. Vynnycky, E. and P.E.M. Fine, *The natural history of tuberculosis: the implications of age-dependent risks of disease and the role of reinfection.* Epidemiology and Infection, 1997. **119**: p. 183-201.
4. Sloot, R., et al., *Risk of tuberculosis after recent exposure. A 10-year follow-up study of contacts in Amsterdam.* Am J Respir Crit Care Med, 2014. **190**(9): p. 1044-52.
5. Sutherland, I., E. Svandova, and S. Radhakrishna, *The development of clinical tuberculosis following infection with tubercle bacilli.* Tubercle, 1982. **62**(4): p. 255-68.
6. Dye, C., et al., *Prospects for worldwide tuberculosis control under the WHO DOTS strategy. Directly observed short-course therapy.* Lancet, 1998. **352**(9144): p. 1886-91.
7. Kunkel, A., et al., *Smear positivity in paediatric and adult tuberculosis: systematic review and meta-analysis.* BMC Infect Dis, 2016. **16**: p. 282.
8. Colebunders, R. and I. Bastian, *A review of the diagnosis and treatment of smear-negative pulmonary tuberculosis.* Int J Tuberc Lung Dis, 2000. **4**(2): p. 97-107.
9. Alpert, P.L., et al., *A prospective study of tuberculosis and human immunodeficiency virus infection: clinical manifestations and factors associated with survival.* Clin Infect Dis, 1997. **24**(4): p. 661-8.
10. Behr, M.A., et al., *Transmission of Mycobacterium tuberculosis from patients smear-negative for acid-fast bacilli.* Lancet, 1999. **353**(9151): p. 444-9.
11. Tostmann, A., et al., *Tuberculosis transmission by patients with smear-negative pulmonary tuberculosis in a large cohort in the Netherlands.* Clin Infect Dis, 2008. **47**(9): p. 1135-42.
12. Ferebee, S., *Controlled chemoprophylaxis trials in tuberculosis a general review.* Advances in Tuberculosis Research, 1970. **17**: p. 28-106.
13. Corbett, E.L., et al., *The growing burden of tuberculosis: global trends and interactions with the HIV epidemic.* Archives of Internal Medicine, 2003. **163**.
14. Tiemersma, E.W., et al., *Natural history of tuberculosis: duration and fatality of untreated pulmonary tuberculosis in HIV negative patients: a systematic review.* PLoS One, 2011. **6**(4): p. e17601.
15. Gagneux, S., et al., *The competitive cost of antibiotic resistance in Mycobacterium tuberculosis.* Science, 2006. **312**(5782): p. 1944-6.
16. Lew, W., et al., *Initial drug resistance and tuberculosis treatment outcomes: systematic review and meta-analysis.* Ann Intern Med, 2008. **149**(2): p. 123-34.
17. Stover, J., R. McKinnon, and B. Winfrey, *Spectrum: a model platform for linking maternal and child survival interventions with AIDS, family planning and demographic projections.* Int J Epidemiol, 2010. **39 Suppl 1**: p. i7-10.
18. UN Population Division, *World Population Prospects, the 2015 Revision*. 2015.
19. Schenzle, D., *An age-structred model of pre and post vaccination measles transmission.* Journal of Mathematics Applied in Medicine and Biology, 1984. **1**.
20. Department of Health South Africa and South African National AIDS Council. *South Africa's National Strategic Plan for HIV, TB and STIs 2017-2022*. 2017; Available from: http://sanac.org.za/wp-content/uploads/2017/05/NSP_FullDocument_FINAL.pdf.
21. World Health Organization, *Systematic screening for active tuberculosis. Principles and recommendations*. 2013, Geneva: WHO.
22. World Health Organization, *WHO Treatment Notifications Database*. 2016: Geneva.

23. Department of Health South Africa. *National tuberculosis management guidelines*. 2014; Available from: http://www.tbonline.info/media/uploads/documents/ntcp_adult_tb-guidelines-27.5.2014.pdf.
24. McCarthy, K., et al., *What happens after a negative test for tuberculosis? Evaluating adherence to TB diagnostic algorithms in South African primary health clinics.* J AIDS, 2016. **71**(5).
25. MacPherson, P., et al., *Pre-treatment loss to follow-up in tuberculosis patients in low- and lower-middle-income countries and high-burden countries: a systematic review and meta-analysis.* Bull World Health Organ, 2014. **92**(2).
26. Churchyard, G.J., et al., *Xpert MTB/RIF versus sputum microscopy as the initial diagnostic test for tuberculosis: a cluster-randomised trial embedded in South African roll-out of Xpert MTB/RIF.* Lancet Glob Health, 2015. **3**(8).
27. World Health Organization, *WHO Treatment Outcomes Database*. 2016: Geneva.
28. World Health Organization. *WHO treatment guidelines for drug-resistant tuberculosis, 2016 update*. 2016; Available from: http://apps.who.int/iris/bitstream/handle/10665/250125/9789241549639-eng.pdf;jsessionid=E7A3B2A7C1806A4C11024C3D695E9E44?sequence=1.
29. Africa, D.D.-R.T.N.D.o.H.S. *Introduction of new drugs, drug regimens and management for drug-resistant TB in South Africa: policy framework. 11th edition*. 2015; Available from: http://www.tbonline.info/media/uploads/documents/policy_framework_ver_20150608.pdf.
30. Shisana, O., et al., *South African National HIV Prevalence, Incidence and Beahviour Survey, 2012*. 2014: Cape Town, South Africa.
31. UN AIDS, *HIV estimates with uncertianty bounds 1990-2016*. 2016.
32. World Health Organization. *WHO TB Burden Estimates*. 2016; Available from: http://www.who.int/tb/country/data/download/en/.
33. Nanoo, A., et al., *Nationwide and regional incidence of microbiologically confirmed pulmonary tuberculosis in South Africa, 2004-12: a time series analysis.* Lancet Infect Dis, 2015. **15**(9).
